# Weakened resilience of benthic microbial communities in the face of climate change

**DOI:** 10.1038/s43705-022-00104-9

**Published:** 2022-03-08

**Authors:** Laura Seidel, Marcelo Ketzer, Elias Broman, Sina Shahabi-Ghahfarokhi, Mahboubeh Rahmati-Abkenar, Stephanie Turner, Magnus Ståhle, Kristofer Bergström, Lokeshwaran Manoharan, Ashfaq Ali, Anders Forsman, Samuel Hylander, Mark Dopson

**Affiliations:** 1 Centre for ecology and evolution in microbial model systems (EEMiS), Linnaeus University, Kalmar, Sweden; 2 Department of Biology and Environmental Science, Linnaeus University, Kalmar, Sweden; 3 Department of Ecology, Environment and Plant Sciences, Stockholm University, Stockholm, Sweden; 4 National Bioinformatics Infrastructure Sweden (NBIS), SciLifeLab, Division of Occupational and Environmental Medicine, Department of Laboratory Medicine, Lund University, Lund, Sweden; 5 National Bioinformatics Infrastructure Sweden (NBIS), SciLifeLab, Department of Immunotechnology, Lund University, Lund, Sweden

**Keywords:** Climate-change impacts, Microbial ecology, Climate-change ecology, Transcriptomics

## Abstract

Increased ocean temperature associated with climate change is especially intensified in coastal areas and its influence on microbial communities and biogeochemical cycling is poorly understood. In this study, we sampled a Baltic Sea bay that has undergone 50 years of warmer temperatures similar to RCP5-8.5 predictions due to cooling water release from a nuclear power plant. The system demonstrated reduced oxygen concentrations, decreased anaerobic electron acceptors, and higher rates of sulfate reduction. Chemical analyses, 16S rRNA gene amplicons, and RNA transcripts all supported sediment anaerobic reactions occurring closer to the sediment-water interface. This resulted in higher microbial diversities and raised sulfate reduction and methanogenesis transcripts, also supporting increased production of toxic sulfide and the greenhouse gas methane closer to the sediment surface, with possible release to oxygen deficient waters. RNA transcripts supported prolonged periods of cyanobacterial bloom that may result in increased climate change related coastal anoxia. Finally, while metatranscriptomics suggested increased energy production in the heated bay, a large number of stress transcripts indicated the communities had not adapted to the increased temperature and had weakened resilience. The results point to a potential feedback loop, whereby increased temperatures may amplify negative effects at the base of coastal biochemical cycling.

## Introduction

Climate change related increases in average surface temperatures [1] and extreme weather events [2] has affected the global oceans [1], resulting in higher CO_2_ concentrations and the concomitant acidification, increased salinity, stratification, de-oxygenation, and rising sea levels [3, 4]. Ocean temperatures are predicted to further rise by up to 2.0 °C down to 100 m in depth [5] and European seas have increased by ~0.01 °C per year since 1860 [6]. Microbes are central to marine energy and nutrient cycles and changes in their community structure and ability to respond to warming will have profound effects on global biogeochemistry and ecosystems. How species diversity responds to these changes might depend on the ecosystem, scales, climate, and organisms considered [7]. In general, experimental warming predicts a reduction of local richness across terrestrial and aquatic ecosystems by 8.9% [7]. However, many global warming predictions use short time and fixed temperature laboratory studies that make it difficult to predict effects in long-term, naturally fluctuating systems [8].

The Baltic Sea is one of the largest brackish-water areas worldwide and is affected by anthropogenic nutrient loading that leads to accelerated eutrophication effects including higher biomass production and increased oxygen consumption that have resulted in a ten-fold expansion of hypoxic (<2 mg/L O_2_) dead zones within the last century [9–11]. Increased temperature accelerates hypoxia by decreasing the oxygen solubility [4] that has accounted for 15% of the total oxygen decline [12] along with higher microbial metabolic rates further increasing oxygen loss [4, 13]. Shallow coastal waters (0–200 m) are more sensitive to atmospheric CO_2_ levels [14] and heat transfer to sediments will be more rapid, likely leading to longer periods of seasonal hypoxia [15]. At higher latitudes, seasonal changes play an important role on the ecosystem structure and nutrient cycling within coastal waters [16] including sediment organic matter (OM) concentrations [17]. Increased temperature and therefore perturbed seasonality could result in a decreased ability of the biological pump to remove carbon from the atmosphere [18]. Microbes in marine sediments play a key role through mineralizing organic matter that removes atmospheric carbon and by recycling nutrients for primary producers [19]. In addition, due to their short generation time coupled with changes in distribution and activity periods (phenological shifts) they are some of the first responders to climate change [20]. However, how these fluctuations will amplify effects through the food web of coastal sediment communities remains poorly understood [21].

Warm water has been discharged for the past 50 years from a nuclear power plant into a Baltic Sea bay, acting as a natural laboratory that can be compared to a non-impacted control bay. The study system serves as a unique opportunity to investigate potential future changes of global warming within a natural fluctuating system, while the control bay was unaffected and represents the contemporary conditions. These types of systems also provide the opportunity to observe coastal climate change effects. The intake for the cooling was from nearby open coastal water at 16–18 m below the sea surface that is heated up to ~10 °C above the ambient water temperature while it cools down the reactors, without being in direct contact with the reactors. This results in an average temperature increase in the heated bay within the predicted range for the RCP5-8.5 (Representative Concentration Pathway) scenario by the year 2100 of 3.3–5.7 °C [22]. However, this predicted temperature increase may also occur in less extreme RCP scenarios in northern hemisphere coastal waters.

In this study, bottom sediment was collected on four occasions in 2018–2019 at three points within each bay generating geochemical parameters (*n* = 9 per bay per sampling). In addition, 16S rRNA gene amplicon (*n* = 9 per bay per sampling) and community RNA transcript (*n* = 3 per bay per sampling; Table S1) data were generated for these samples. In this study, we investigated: (1) how does 50 years of warming affect microbial community diversity and structure; (2) how and to what degree does increased warming influence microbial energy and nutrient cycling; (3) if the warming resulted in phenological shifts; and 4) what consequences do these effects have in the face of future climate change.

## Results and discussion

### Geochemical parameters

**Fig. 1:**
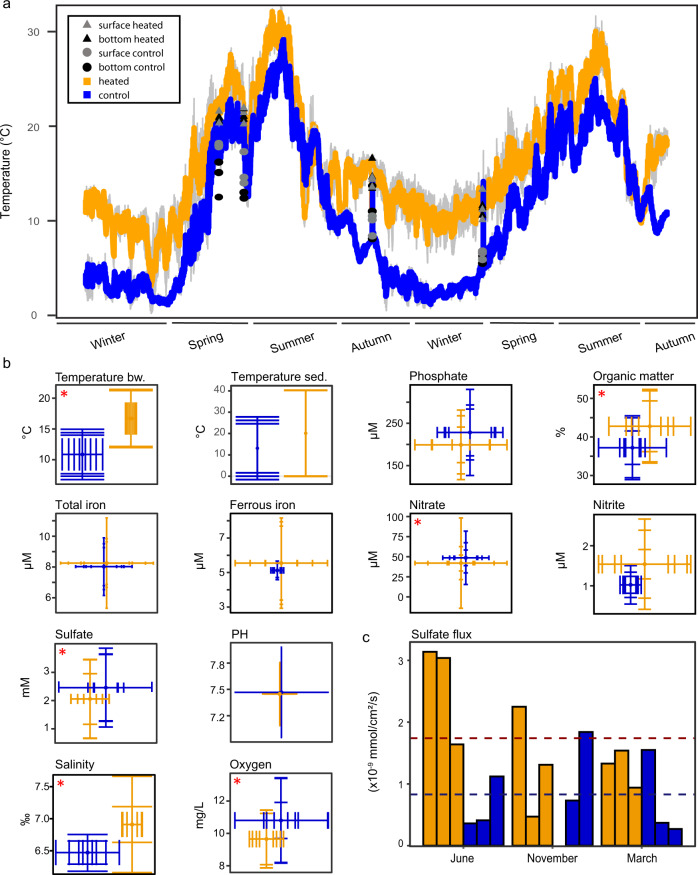
Spatiotemporal variation in geochemical parameters in the heated and control bays. **a** Temperature measured within the heated (orange) and control (blue) bays using HOBO data loggers at three different sampling sites (1 m below the surface) between December 2017 and November 2019. Standard deviations (*n* = 3) between the sampling sites in each bay are shown in light gray. Surface (gray) for heated bay (▲) and control bay (●) and bottom (black) water temperatures were also measured at each sampling time. **b** Mean sediment pore water (0–1 cm) and bottom water geochemical parameters from the heated (orange) and control (blue) bays are indicated with dots. Temperature, oxygen, and salinity were measured on bottom water with the other environmental variables measured on pore water from the collected sediments. Vertical standard deviations (s.d.) show spatial variation over sampling sites within each bay (*n* = 3) while horizontal deviations (s.d.) represent the temporal variation over sampling occasions (*n* = 4). Temperature of the sediment were taken June 2018 (heated bay 17.5–31 cm depth and control bay 23.5–33 cm depth). Red asterisks indicate significant changes between the bays (Supplementary Table 2). **c** Downward sulfate diffusive fluxes at 6 cm sediment depth for three sampling points across the heated (orange) and control (blue) bays at three different sampling points (June 2018, November 2018, and March 2019). Dashed lines show the average sulfate fluxes for the heated (dark orange; 1.74 × 10^–9^ mmol/cm^2^/s) and control (dark blue; 0.83 × 10^–9^ mmol/cm^2^/s) bays.

The heated bay was on average 5.1 °C (mean±s.d. control bay 10.9 ± 3.6 °C, heated bay 16.8 ± 4.3 °C) warmer than the control bay with greater temperature differences in October to February and little or no difference during June to August 2018 (Fig. 1). The surface and bottom water temperatures were not statistically different in the heated bay (General linear model (GLM), ANOVA, *F*_9,62_ = 0.01, *p* = 0.929) that was likely due to mixing of the water column by the discharge water while a thermocline was observed with varying intensity in the control bay (*F*_9,62_ = 27.03, *p* < 0.0001). While these temperature increases were on the outer range predicted for 2100 (with 3.3–5.7 °C by year 2100 according to SSP5-8.5 [22]), they represent relevant temperature increases for coastal areas [23, 24].

Spatiotemporal differences in bio- and geochemical parameters at shallow depth below the seafloor (0–1 cm) were observed between the two bays (Fig. 1 and Table S2). Oxygen concentrations in water decreased with higher temperatures [13] while microbial metabolic rates increased [25], likely causing the observed lower oxygen concentration in the heated bay (mean ± s.d. = 9.76 ± 4.3) compared to the control bay (10.76 ± 3.6 mg/L; GLM, ANOVA, *F*_*17,49*_ = 9.84, *p* = 0.0028). However, the bottom waters were not hypoxic as the sampling sites were likely too shallow (1.2–4.9 m; Supplementary Table 1) allowing permanent reoxygenation. The mean sediment organic matter content after loss on ignition analysis of 42.3 ± 8.9% in the heated bay was significantly higher than the control bay (37.2 ± 6.9%, ANOVA, *F*_*17,49*_ = 11.39, *p* = 0.0144) potentially due to elevated growth rates and higher primary production as a result of e.g. increased temperatures plus necromass from algal blooms sinking to the sediment. The increased necromass led to higher use of electron donors that likely supported anaerobic nitrate reduction and was verified by an increased but highly variable mean sediment porewater nitrite concentration of 1.59 ± 1.5 µM in the heated bay compared to 0.98 ± 0.42 µM in the control bay with a concomitant significantly lower nitrate concentration in the heated versus control bay (42.85 ± 36.6 and 48.16 ± 24.1 µM; *F*_*17,49*_ = 4.96, *p* = 0.03). In addition, the mean ferrous iron concentration, likely from ferric reduction, was higher but also varied largely over the year in the heated versus control bay (5.56 ± 3.2 and 5.12 ± 0.5 µM). Higher sulfate reduction rates likely resulted in significantly lower sulfate concentration (2.04 ± 1.2 mM) in the heated bay 0–1 cm sediment depth compared to the control bay (2.52 ± 1.3 mM; *F*_*17,49*_ = 13.95, *p* = 0.005). The mean phosphate concentration was also lower in the heated bay (201.3 ± 109.4 µM heated bay and 223.1 ± 103.7 µM control bay) potentially due to increased microbial activity consuming phosphate for growth, precipitation of ferric phosphate [26], phosphate input in the control bay from e.g. Cyanobacteria (Fig. S2), and variations in anthropogenic phosphorus input into the two bays [27]. In summary, these data supported increased organic matter accumulation in the heated bay sediments linked to a decrease in electron acceptors associated with anaerobic energy conservation.

### Sulfate flux

The geochemical data (Fig. 1) corroborated previous findings that sulfate concentration and flux in sediment pores are primarily controlled by the rate of organic matter mineralization via sulfate reduction [28], with higher temperatures leading to elevated reduction rates and a thinning/shallowing of the sulfate reduction zone towards the sediment-water interface. For instance, pore water samples at 6 cm below the seafloor in the heated bay showed lower average sulfate concentrations and a two-fold increase in the average sulfate flux in relation to the control bay (2.92 versus 4.01 mM and 1.74 versus 0.83 × 10^−9^ mmol/cm^2^/s, respectively). A thinner/shallower sulfate reduction zone also implied that methanogenesis, which mostly occurs after sulfate in pores is consumed [29], will occur closer to the sediment-water interface and facilitate methane emissions from the seafloor. In summary, prolonged warming will likely lead to a compression of geochemical zones with sulfate reduction occurring closer to the sediment surface, which potentially facilitates sulfide release from the sediment. Therefore, this thinning/shallowing of anaerobic energy conservation may result in exacerbating the effects of climate change by initiating a negative feedback loop with increased dead zones.

### Microbial diversities

**Fig. 2:**
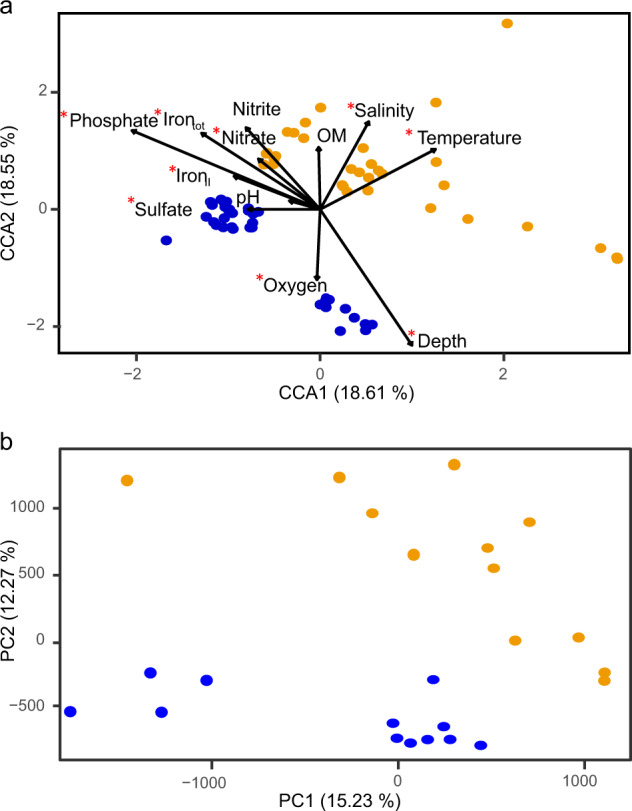
16S rRNA gene amplicon ASVs and RNA transcript microbial beta diversities. **a** Canonical correspondence analysis plot of microbial communities (based on relative abundance of ASVs) with explanatory environmental variables (vectors) for the four different sampling time points in the heated (orange) and control (blue) bays. Chemical parameters were measured in the pore water of the 0–1 cm sediment, organic matter (OM) was measured from the sediment, and temperature, salinity, and oxygen were measured in the bottom water. Red asterisks indicate parameters that significantly explain the microbial community variation (Supplementary Table 2). **b** Principal component analysis based on VST transformed RNA transcript counts filtered for at least five reads within at least three samples for the heated bay (orange; *n* = 12) and the control bay (blue; *n* = 12).

**Fig. 3:**
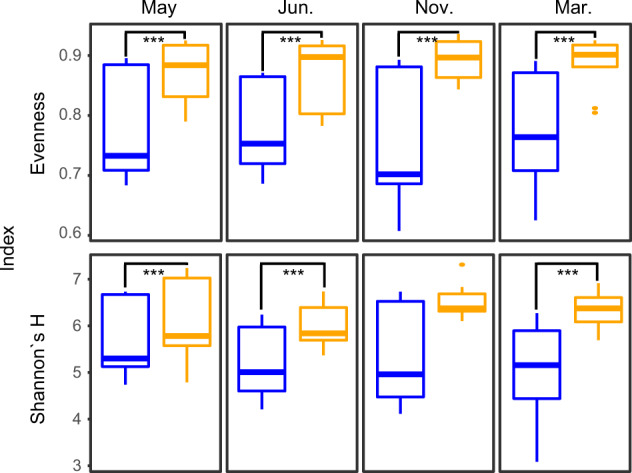
16S rRNA gene amplicon ASV diversity indices. Shannon´s H index and evenness diversity indices for the heated (orange) and control (blue) bays for each sampling month. Linear regression model for testing significant differences between bays (*n* = 36) with pairwise comparison for testing differences between bays on each sampling month (*n* = 9 per bay) was used (Supplementary Table 2). Asterisks indicate significant differences between diversity of microbial communities at each sampling month; ***p ≤ 0.001. Shown are the minimum relative abundance, first quartile (25%), third quartile (75%), and the maximum relative abundance. The upper and lower whisker extends from the hinge at most 1.5*IQR (interquartile range), data beyond are plotted individually and marked as outliers. Outliers are given as dots.

**Fig. 4:**
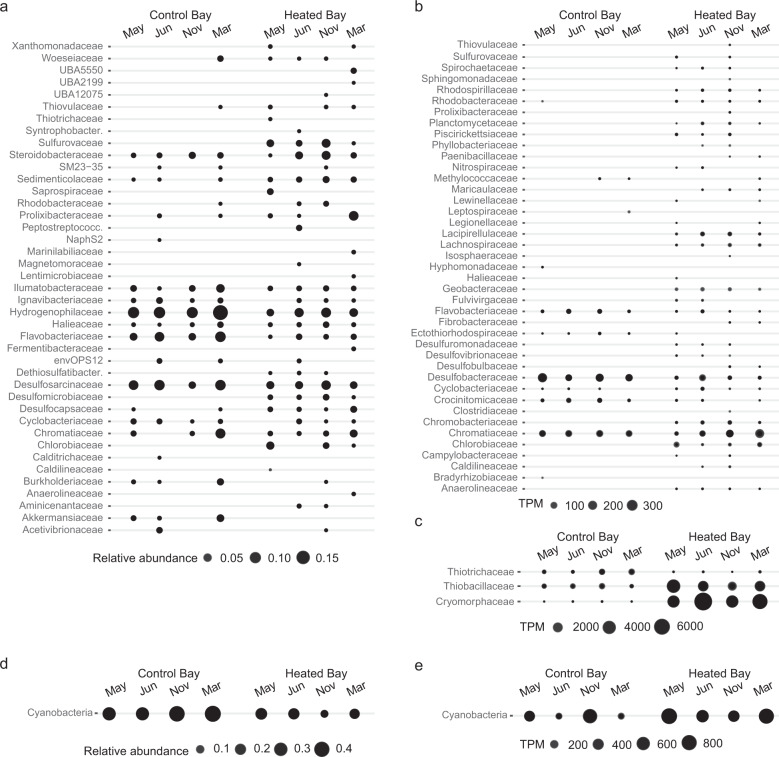
Abundances of ASVs and RNA transcripts in the heated and control bay for major taxa. **a** Balloon plot with significant (adjusted *p*-value < 0.05) differentially abundant ASVs with >0.5% abundance annotated on family level. The abundance was calculated on at least 0.5% relative abundance in a sample. The control bay with month May, June, and November 2018 plus March 2019 are shown on the left while the heated (temperature-affected) bay with sampling month are shown on the right. Shown are the sum of replicates (*n* = 3) and sampling sites (*n* = 3) per bay on the four different sampling occasions with circle sizes showing the abundance of families at each time point and location. **b** Balloon plot of top 100 TPM per sample of genes filtered for significant (adjusted *p*-values < 0.05) differential expression within the bacteria annotated on known family level (Cyanobacteria and unknown on family level filtered out) on the *y*-axis. The control bay with month May, June, and November 2018 plus March 2019 plotted on the left while the heated bay (temperature-affected) with sampling month are shown on the right. Shown are the TPM sum of sampling sites as replicates (*n* = 3) per bay on the four differe*n*t sampling occasions; circle sizes show the TPM value of families at each time point and location. **c** Additional balloon plot of the data from **b** of the sum of TPM of the families Thiotrichaceae, Thiobacilliaceae, and Cryomorphaceae of sampling sites as replicates (*n* = 3) per bay on the four different sampling occasions from the top 100 TPMs per sample. **d** Significant (adjusted *p*-values < 0.05) differential abundant ASVs related to the phylum Cyanobacteria. **e** Sum of top 100 TPM per sample of genes filtered for significant (adjusted *p*-values *p* < 0.05) differential expression related to Cyanobacteria.

Sediment microorganisms are commonly stratified according to their electron acceptor requirements for energy conservation from e.g. reduction of oxygen, nitrate, ferric iron, sulfate, and carbon dioxide [29] although this distribution is more complex in coastal areas where other environmental factors (including spatial heterogeneity plus episodic input of organic carbon or photosynthesis) influence the stratification [30]. The decreased oxygen concentration in the heated bay may be a driver of microbial community clusters [31] and seasonal temperature influences on microbial activities (Fig. 2 and Table S2). Within the dataset, doubletons made <4% of the overall relative abundance of the community and did not change the diversity differences of the bays (Fig. S3 and Table S2). The amplicon sequencing variant (ASV) Shannon´s H indices (6.22 ± 0.6 and 5.28 ± 0.9) and evenness (0.88 ± 0.04 and 0.77 ± 0.09) were significantly higher (ANOVA, Shannon´s H, *F*_*17,49*_ = 59.78, *p* < 0.001 and evenness, *F*_*17,49*_ = 234.93, *p* < 0.001) in the heated bay that had warmer and more stable (both in space and time) temperatures compared to the control bay (Fig. 3 and Table S2 and Fig. S3). The overall higher number of species found (Chao1, 1306.22 ± 566.01 heated bay and 1096.722 ± 496.11 control bay; Fig. S3) within the heated bay was contrary to the classical niche theory of spatial environmental heterogeneity harboring more species due to niche availability [32]. This was also counter to the expectation that the larger temperature fluctuations in the control bay and concomitant coexistence of multiple species with different optimum growth temperatures that become dominant at various times of the year (Figs. 3, 4 and Fig. S4) would result in an overall higher microbial diversity [7]. The data supported that the thinner geochemical zones, increased respiration rates shown by augmented sulfate flux, and consumption of anaerobic electron acceptors selected for a greater microbial diversity in the 0–1 cm below seafloor heated bay sediment.

### Microbial communities

A comparison of the 16S rRNA gene amplicon and RNA transcript data suggested no noteworthy influence of the cooling water intake of the more open Baltic Sea into the heated bay. For example, the dominant species annotated from the RNA transcripts in the heated bay sediment could also be found within the control bay at lower numbers (e.g. *Thiobacillus*). Additionally, comparing data from benthic and open water communities within the Baltic Sea showed evidence that the microbes from in-flowing cooling water containing open Baltic Sea water did not noticeably influence the overall sediment microbial community composition [11, 33, 34].

Canonical correspondence analysis (CCA) supported that the microbial community compositions were different between the two bays (PERMANOVA, *F* = 13.99, *p* = 0.001, Fig. 2 and Table S2). In addition to temperature, the main drivers separating the communities in the two bays were depth and the sampling location (Fig. 2). Further, discussion of the validity of the communities within the heated bay despite input of microbes from the cooling system water is provided in the method section of the Supplemental Information. The most abundant 16S rRNA gene ASVs from both bays aligned within the Proteobacteria (23.2% and 17.6%), Cyanobacteria (20.9% and 45.9%), and Bacteroidota (16.4% and 11.7%) that matched previous results from Baltic Sea sediments [11]. Dominant taxa in the control bay included chemolithotrophic or mixotrophic Hydrogenophilaceae [35], the oxygen positively correlated Flavobacteriaceae, and the facultative anaerobic Ignavibacteriaceae [36] compared to a more even distribution of a greater number of taxa including the sulfate reducing Desulfomicrobiaceae [37] and Desulfocapsaceae [38] plus the anoxic sediment family Woesiaceae [39] in the heated bay (Fig. 4 and Tables S3–S4). The microbial communities were highly affected by the generally lower oxygen availability in the heated bay with the control bay following seasonal patterns with warmer summer temperatures favoring lower oxygen conditions in the bottom sediments. However, the heated bay also showed increased relative abundance over the year of some aerobic organic matter degrading bacteria including ASVs aligning with e.g. the *Arenimonas* genus (Fig. S4) that may have contributed to a higher and constant oxygen consumption [40]. This likely resulted in thinner geochemical zones, a switch to anaerobic respiration closer to the sediment-water interface, and a greater number of niches for anaerobic bacteria in the sampled top 1 cm below the seafloor. After oxygen was depleted, nitrate was potentially reduced by mainly Proteobacteria including Steroidobacteraceae [41] and Sulfurovaceae [42] leading to higher nitrite concentrations (Figs. 1, 2 and 4). Iron reduction in the heated bay sediments may have been mediated by the significantly higher abundant Rhodobacteraceae [43] (differential abundance analysis, *p* < 0.05; Table S4) or by sulfate reducing bacteria also capable of ferric reduction [44] (*p* < 0.05; Figs. 1, 2 and 4). The lower sulfate concentration in the heated bay sediment over the whole year was likely associated with the concomitant higher abundance of sulfate reducing taxa [30] including *Desulforhopalus*, *Desulfofustis*, and *Desulfomicrobium* that only occurred in the heated bay (Fig. S4). Sulfate reduction results in reduced sulfur that was likely oxidized by the increased relative abundance over all sampling times in the heated bay of Chromatiaceae, *Candidatus* Thiodiazotropha, and the positively correlated with increasing temperature Sulfurovaceae family (all *p* < 0.05; Fig. 4, Fig. S4, and Tables S3–S5). Cyanobacteria had a high relative abundance in both bays with a statistically higher relative abundance in the control bay (*p* < 0.05; Table S4). While resolution at higher taxonomic levels was limited, Chroococcales was highly abundant at one sampling site in the heated bay compared to Oscillatoriales and Synecchocales in the control bay (Fig. S5). The high amounts of Cyanobacteria in the sediment of both bays increased organic carbon that consumes oxygen during mineralization and is suggested to lead to coastal hypoxia in the Baltic Sea as a whole [45]. In contrast, the shallow depth may have permitted photosynthesis by Cyanobacteria on the sediment surface that would produce oxygen. In summary, condensed geochemical layers in the heated bay led to selection of a high diversity of ASVs in the 0–1 cm sediment layer predominantly aligning with taxa characterized as anaerobic and cycling sulfur compounds.

### RNA transcript based activities

The majority of the RNA transcripts originated from Bacteria (75.7%) followed by Eukaryotes (6.7%), Archaea (1.8%), and viruses (<0.001%; Fig. S6). The Bacteria transcripts generated 2268 unique genes from 404 families (Fig. S7 and Table S6). Principle component analysis (PCA) based upon RNA transcripts gave a very similar distribution between the bays as that observed for the 16S rRNA gene amplicon data (Fig. 2). Significantly different RNA transcripts with an annotated function (DESeq2 differential expression analysis; adjusted *p* < 0.05; Fig. S7 and Table S7) also showed fewer transcripts attributed to more diverse families within the heated bay compared to higher transcript numbers to fewer, more dominant families in the control bay (Fig. 4). This further supported the data as representative of the sediment microbial communities and activities.

High numbers of RNA transcripts in both bays were annotated as taxa involved in sulfur cycling (Fig. 4). These included several sulfate reducing microbial families in the heated bay such as Desulfuromonadaceae, Desulfovibrionaceae, Desulfobulbaceae, and Desulfobacteraceae compared to fewer diverse microbial sulfate reducing families, but with a higher activity that included the Desulfobacteraceae in the control bay. The generated sulfide was likely oxidized by a broad range of taxa with different relative counts of RNA transcripts for e.g. Thiobacillaceae that can oxidize sulfur compounds in both oxic and anoxic conditions [46] predominantly identified in the heated bay and aerobic Thiotrichaeceae [47] largely in the control bay. In addition, the Chromatiaceae family was both present and active in both bays suggesting anoxic sulfide oxidation [48]. A further difference in RNA transcripts was observed for the phylum Cyanobacteria with high counts suggesting all year round activity in the heated bay compared to seasonal blooms of activity in the control bay (Fig. 4). While Cyanobacteria had a statistically higher 16S rRNA gene amplicon relative abundance in the control bay, the RNA transcripts showed temporal peaks in activity in the control bay, compared to all year-round activity in the heated bay.

The data support that future prolonged warming related to climate change may result in a phenological shift with longer periods of Cyanobacteria activity leading to anoxic conditions in the sediment surface [49]. In addition, both bays exhibited evidence of cryptic sulfur cycling as has been observed in nearby Baltic Sea sediments [11].

### Metabolic responses

**Fig. 5:**
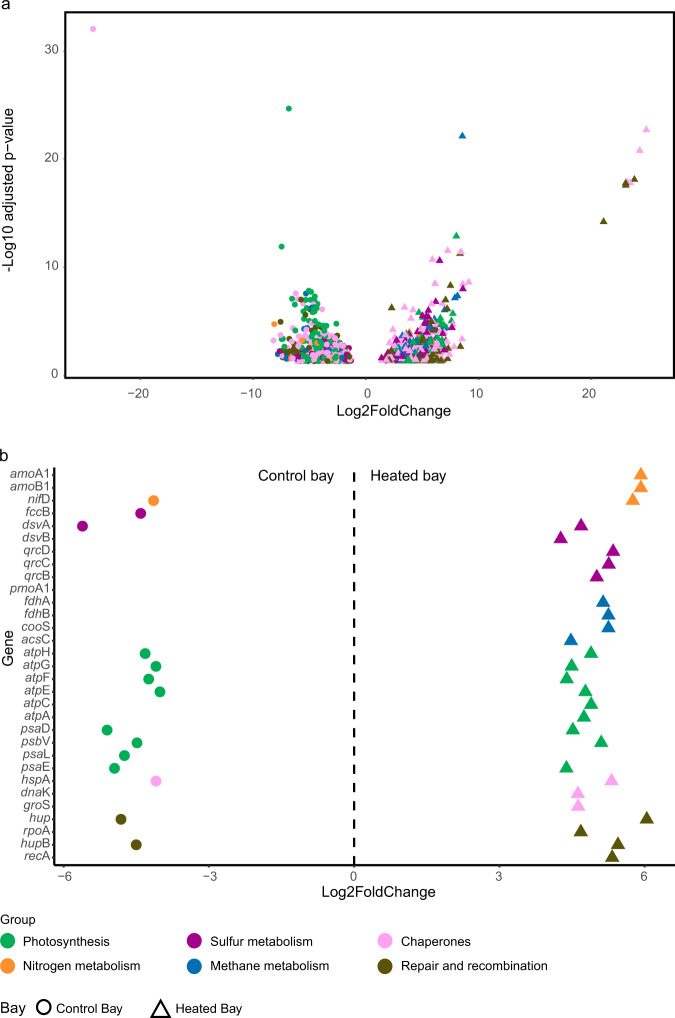
Metabolic responses of differential expressed genes. **a** Volcano plot of significantly differential expressed genes (adjusted *p*-value < 0.05) selected as related to energy metabolism, stress, and repair KEGG categories. LFC are shown on the *x*-axis while the –log10 adjusted *p*-values are shown on the *y*-axis for differential RNA transcripts in the control (●) and heated (▲) bays. **b** Selected RNA transcripts with an LFC > 4 for energy metabolism, stress, and repair. LFC is shown on the *x*-axis while genes are shown on the *y*-axis for the control (*n* = 12; left side) and heated (*n* = 12; right side) bays. The data represent mean LFCs of RNA transcripts of genes as separately calculated for each bay.

Comparison of highly statistically significant RNA transcripts (log fold change (LFC) ≥ 4 or ≥ −4 and *p* < 0.05; Fig. 5 and Table S7) identified 595 and 553 differentially abundant genes in the heated and control bays, respectively. Of these, differences in RNA transcripts between the two bays were annotated as related to nitrogen (5 and 8 genes), sulfur (13 and 10), and methane metabolism (21 and 23), photosynthesis (36 and 42), chaperones (27 and 26), and repair (20 and 18; Fig. 5). Transcripts with a high LFC annotated as involved in nitrogen metabolism lacked genes coding for dissimilatory nitrogen metabolism as both microbial communities were likely cycling nitrogen compounds. Transcripts coding for oxygen consuming methanotrophy (*pmo*A1, mean LFC 3.18 heated bay vs. −3.47 control bay) and sulfur oxidation (*fcc*B, 2.63 vs. −4.4) processes had higher LFC values supporting the overall higher oxygen concentration in the control bay [50]. In contrast, transcripts for dissimilatory sulfate reduction (*dsv*B (4.26 vs. −2.75), *apr*AB (3.9 vs. −3.53, 3.8 vs. −3.09), and *qrc*BCD (5.01, 5.25, 5.35) were higher in the heated bay that supported the geochemical and 16S rRNA gene amplicon data of compressed geochemical zones. Further support for the compressed geochemical zones included transcripts annotated as involved in methanogenesis [51] (*fdh*AB (5.1, 5.25), *coo*S (5.25 vs. −2.89), and *acs*AC (4.81 vs. −2.36, 4.47 vs. −3.57) mainly identified in the heated bay along with the key *mcr*BC genes (Table S6) attributed to Archaea that were mainly identified in the heated bay. Energy conservation processes such as the ATP-synthase with higher transcripts in the heated bay (particularly evident at one sampling site) supported that increased temperature due to climate change increases productivity [25]. In contrast, RNA transcripts related to photosynthesis genes (PSI and PSII) had generally higher numbers within the control bay, suggesting the dominance of cyanobacterial blooms during early summer and autumn [26] compared to the all year round activity of Cyanobacteria in the heated bay (Fig. 4 and Fig. S8). The increased RNA transcripts for photosynthesis genes suggested the Cyanobacteria were on the sediment surface at a depth where light penetrates. Further analysis identified a large number of chaperone transcripts with a LFC > 4 that were predominantly identified in the heated bay and included heat stress proteins such as *hsp*A (5.31 vs. −4.09), *dna*K (4.62 vs. −3.59), *gro*S (4.62 vs. −3.46), and *ibp*A (3.99 vs. −3.59) [52]. However, genes coding for stress proteins were also identified in the control bay, albeit with much fewer transcripts and partly during the summer when the temperature was similar to the heated bay (Fig. S8). In addition, a similar trend of RNA transcripts from the DNA repair and recombination proteins were identified in the heated bay, likely as a response to the described stress [53] (Fig. S8).

The presence of over 500 statistically different RNA transcripts with a LFC ≥ 4 in each bay suggested that large changes had occurred between the respective microbial community activities and that future climate change would profoundly change Baltic Sea sediment communities. The results further supported condensed geochemical zones with a higher metabolic activity of sulfate reduction and methane production in the heated bay. Furthermore, more RNA transcripts associated with ATP production were identified in the heated bay suggesting higher metabolic rates and energy generation. While the heated bay organic matter content supported increased production, this was likely tempered by the additional energy demand to alleviate stress and repair cellular components such that it may not be completely translated into cellular reproduction [54]. The large number of heated bay RNA transcripts related to stress also suggested that while the microbial community had altered, the community members’ temperature optima were below that of the heated bay. Therefore, coastal sediment communities will likely take greater than 50 years to become adapted to the increased temperature such that the increased energy production is fully converted to higher replication rates.

## Conclusions

A strength of this study was that it was carried out in the natural environment with seasonal fluctuations. Despite inherent complications such as potential differences in nutrient inputs into the two bays, this work showed that warmer temperatures in the heated bay over 50 years selected an altered sediment microbial community with decreased seasonal variation, resulting in more stable and diverse sediment communities at 0–1 cm below the seafloor. These included Cyanobacteria that result in a potential prolonged bloom period and increased coastal anoxia connected to climate change. Previous studies suggest warmer temperatures will result in increased energy production [25] and this was also supported in our study with more RNA transcripts associated with energy production and higher organic matter content in the heated bay. However, the stress and repair transcripts suggested the microbes’ temperature optima were below that of the water such that the increased productivity may be tempered and the community’s resilience may be weakened. This could result in a negative feedback loop where the increased warming related effects will lead to an increase in Cyanobacteria production, elevated organic matter degradation, lower oxygen concentrations, and shallowing of geochemical zones with potential methane release to the atmosphere in already oxygen deficient zones that exacerbates the ongoing climate change. However, it remains to be confirmed if such altered microbial processes would occur in coastal sediments worldwide and such studies could be carried out in additional thermally altered areas.

## Materials and methods

### Sampling sites

Sampling was conducted in two Baltic Sea bays near the city of Oskarshamn, Sweden (GPS coordinates for the six sampling sites are given in Table S1). The heated bay has been used as an outlet for cooling water from a nuclear power plant for nearly 50 years and is open on one side to the Baltic Sea. Besides the increased temperatures, other environmental factors are also likely to differ between the bays that could potential influence the results. However, radioactivity is unlikely to be one of these factors, because the levels (bq/kg sediment) associated with the nuclear power plant in the heated bay were lower than or equal to natural background radiation as well as still measurable fallout at our study site from the Chernobyl accident [55]. Yet, to fully evaluate the contributions of all these factors, would require replicated approach that is logistically very challenging and beyond the scope of this contribution. The control bay was not subjected to artificial temperature changes and is not connected to the heated bay in any other way than the open Baltic Sea with a distance of ~1.5 km. More details of the sampling sites and their geochemistry can be found in the supplemental information.

### Sample analysis

Chemistry analysis was conducted on surface and bottom water in situ. A kajak gravity corer was used to sample with three acrylic transparent cores (inner diameter: 7 cm, length: 60 cm) at each sampling location as biological replicates (see Supplementary Table 1 for the sampling depth) giving nine cores per bay and 18 cores per sampling occasion. The 0–1 cm sediment surface was sliced and samples for nucleic acid extractions and chemistry measurements were collected as previously described [26]. DNA from homogenized sediment samples (250 mg) was extracted and PCR amplification and Illumina library preparation was conducted as described in Lindh et al. [56]. RNA extraction from homogenized sediment samples (2 g) was carried and frozen at −80 °C until sending for sequencing. A more detailed description on the sample analysis can be found within the supplemental information.

### Sequencing and statistical analysis

Samples for 16S rRNA analysis were sequenced at the Science for Life Laboratory (SciLifeLab) in Stockholm. The sequences were analyzed using the DADA2 pair-end pipeline (benjjneb.github.io/dada2/index.html) (v. 1.16) on the UPPMAX cluster (Uppsala Multidisciplinary Center for Advanced Computational Science). The final data were analyzed using R [57]. RNA samples were processed and sequenced at the DOE Joint Genome Institute at the Lawrence Berkeley National Laboratory, Berkeley, USA. Quality control filtering was conducted by JGI. A more detailed description of the methods used and the statistical analysis, can be found in the supplemental information within the Material and Method section.

## Supplementary information

Supplemental Material

Table S1

Table S3

Table S4

Table S5

Table S6

Table S7

Table S8

## Data Availability

The supplementary file S6 can be found on https://github.com/laseab/CC_WR. 16 S rRNA gene sequencing data are available on the NCBI database under BioProject PRJNA739524. RNA transcript raw reads are available on the JGI Integrated Microbial Genomes and Microbiomes (IMG) database with the following references JGI proposal ID 503869.

## Code availability

The code used to process the 16 S rRNA, chemistry, and meta-transcriptomic data can be found on https://github.com/laseab/CC_WR.
