# Supplementary material for: Weakened resilience of benthic microbial communities in the face of climate change
Source: ISME Commun. 2022 Mar 8;2:21. doi: 10.1038/s43705-022-00104-9 (PMC9723771; doi:10.1038/s43705-022-00104-9)
Supplement: Supplementary file 1 — Supplemental Material [file 43705_2022_104_MOESM1_ESM.pdf]

Supplementary Information for
Weakened Resilience of Benthic Microbial Communities in the Face of Climate Change.

Laura Seidel*, Marcelo Ketzer, Elias Broman, Sina Shahabi-Ghahfarokhi, Mahboubeh Rahmati-Abkenar, Stephanie Turner, Magnus Ståhle, Kristofer Bergström, Lokeshwaran Manoharan, Ashfaq Ali, Anders Forsman, Samuel Hylander, and Mark Dopson

Laura Seidel
Email: laura.seidel@lnu.se

This PDF file includes:

Material and Methods
Figures S1 to S10
Table Information S1 to S8
Table S2

Material and Methods

Sampling sites

Sampling was conducted in two Baltic Sea bays near the city of Oskarshamn, Sweden (GPS coordinates for the six sampling sites are given in Table S1). The heated bay has been used as an outlet for cooling water from a nuclear power plant for nearly 50 years and is open on one side to the Baltic Sea. The cooling water within the power plant system was heated up to ~10°C above the ambient water temperature (1). The control bay was not subjected to artificial temperature changes and is not connected to the heated bay in any other way than the open Baltic Sea with a distance of approximately 1.5 km. Three sampling sites were chosen within each bay (Fig. S1) and sampling was carried out on four occasions between May 2018 and March 2019 (Table S1).

Temperature tracking in the two bays

Six temperature data loggers (HOBOware, Onset Computer Corporation, USA) were placed at three different sampling sites in each bay from December 2017 until November 2019 (Fig. S1). The sensors were attached to a buoy at 1 m depth below the surface and logged the temperature hourly. The data were read with the Freeware Software for Data loggers & Devices from Onset Computer Corporation (v. 3.7.13). Temperature data was plotted using the ggplot package in R (2, 3). Additionally, temperature loggers were attached to the acrylic cores and were used to measure the sediment temperature at each sampling site in June 2018 (Fig. 1 & Table S1).

Sediment and bottom water sampling and analysis

The pH, temperature, salinity, and oxygen concentrations in the surface and bottom water were measured *in situ* (Multiline™ sensor, WTW™). A kajak gravity corer was used to sample with three acrylic transparent cores (inner diameter: 7 cm, length: 60 cm) at each sampling location as biological replicates (see Supplementary Table 1 for the sampling depth) giving nine cores per bay and 18 cores per sampling occasion. The 0-1 cm sediment surface was sliced and samples for nucleic acid extractions and chemistry measurements were collected as previously described (4) and returned to the laboratory on the same day with additional 50 mL sediment at 6 cm below seafloor and 50 mL bottom water for sulfate flux measurements. Chemistry analysis was conducted from sediment as well as pore water as described in Broman *et al.*(4) except for OM where 2 mL was dried for six days at 40°C instead of three days at 80°C (before loss on ignition analysis). During the analysis of OM (% wt) the dried sediment were ignited at 550°C for 4h in a furnace (OWF 1200, Carbolite) and the percentages are calculated from the loss of weight before and after the process (5). NO_2^- plus NO_3^- were measured using Hach-Lange cuvette tests (LCK339 Nitrate and LCK341 Nitrite; Hach-Lange, Hach®). Sulfate diffusive fluxes at 6 cm below the seafloor was calculated based on sulfate concentrations in pore waters at this depth, temperature, porosity, and salinity. Pore water from the June 2018 and November 2018 sampling campaigns was collected by centrifuging 50 mL of sediment (5800 *g* for 10 min at 4°C) and from the March 2019 campaign by using rhizon samplers (6). The water phase was extracted, filtered (4), and analyzed using the Hach-Lange cuvette test for sulfate (LCK353; Hach-Lange, Hach®). Temperature and salinity were measured for each sampling point and porosity was assumed to be 70% (within the range of uncompacted

mud) (7). The flux was obtained by the Fick´s first law and the effect of porosity on diffusion was considered through a logarithmic equation (8). Details of samples and chemistry parameters are given in Table S1.

Nucleic acids extraction

DNA from homogenized sediment samples (250 mg) was extracted with the DNeasy® PowerSoil Extraction Kit (QIAGEN) according to the manufacturer's guidelines. Extracted DNA was stored at -20°C until PCR amplification and Illumina library preparation. RNA extraction from homogenized sediment samples (2 g) was carried out using the RNeasy® PowerSoil Total RNA Kit (QIAGEN) and phenol/chloroform/isoamyl alcohol method (VWR Life Science). RNA samples were DNase treated twice with the Turbo DNA-free kit (Ambion) and frozen at -80°C until sending for sequencing. DNA and RNA concentrations were measured using a NanoDrop 2000 (Thermo Scientific™) and Qubit® 2.0 (Invitrogen™, Life Technologies Corporation). For preparation of the 16S rRNA gene Illumina library, the PCR primers 341f and 805r and a modified PCR program were used (9). Modifications of the different processes of adding Illumina adapters and indices were performed according to Lindh et al (10).

16S rRNA gene amplicon sequencing and analysis

Samples for 16S rRNA analysis were sequenced at the Science for Life Laboratory (SciLifeLab) in Stockholm on the Illumina MiSeq platform with 2 × 301 bp pair-ends. The sequences were analyzed using the DADA2 pair-end pipeline (benjjneb.github.io/dada2/index.html) (v. 1.16) on the UPPMAX cluster (Uppsala Multidisciplinary Center for Advanced Computational Science). Sequences were trimmed at 290 fw and 230 rv and left trimmed at 21 bp. The error-model was run with a MAX_CONSIST of 30 for the forward and reverse reads and were merged together with a minimum overlapping length of 10 bp with zero mismatches allowance.

The average sequence count after the quality filtering and merging of pair ends was 64164 reads (min. 6417 and max. 170279; Table S8). After removing chimeras, the taxonomy was assigned against the Silva database (v. 132) training set for dada2 (benjjneb.github.io/dada2/training.html). The final data were analyzed using R (3). Packages used within the R environment are stated at the specific analysis step within the methods. A total of 20340 unique ASVs could be detected of which 61 % were either single- or doubletons (12604). Analysis of alpha diversity on a subset of the data without single- or doubletons showed similar patterns of higher diversity and evenness in the heated bay compared to the control bay (Fig. S3). Therefore, analysis was continued on the whole dataset. The first analysis showed high abundances of Cyanobacteria (37% of ASVs over 0.5% relative abundance) that were mainly unclassified (67% on family level) on lower taxonomic levels. The pattern of the analysis did not show notable changes with or without Cyanobacteria in the dataset and therefore, Cyanobacteria were filtered out for further analysis to obtain a clearer overview on the other sediment bacteria within the bays (Fig.

S2). As the 16S rRNA gene sequencing data are relative abundances, the data are compositional (11) and follow Aitchison geometry due to the inherent closed structure of the data (12). Thus, in order to perform analysis of variance the raw data should be first transformed.

Community transcriptome sequencing and analysis

RNA samples were processed and sequenced at the DOE Joint Genome Institute at the Lawrence Berkeley National Laboratory, Berkeley, USA. Ribo-Zero Bacteria and Plant-Leaf rRNA Removal Kits from Illumina in a 50:50 ratio were used for rRNA depletion. Samples were prepared with the TruSeq Stranded Total RNA HT sample preparation kit following the manufacturer's protocol with 500 ng total RNA starting material per sample and eight cycles of PCR for library preparation. The library was quantified using the next-generation sequencing library qPCR kit from KAPA Biosystem (Roche) on a Roche LightCycler 480 real-time PCR. The quantified library was multiplexed and pooled for sequencing. The samples were sequenced on an Illumina NovaSeq 6000 sequencing platform using NovaSeq XP v1 reagent kits (Illumina) and a S4 flow cell (Illumina) giving sequences with 2 × 151 bp read length.

Quality control filtering was conducted by JGI and briefly, contaminants were removed using BBDuk (13) (v. 38.75) as well as trimming reads that contained "N" bases and reads with an average quality score across reads less than 10. The program BBMap (13) was used to filter and remove human, cat, dog, and mouse reads referenced at 93% identity, as well as reads aligned to common microbial contaminants and reads containing known spike-ins. Finally, ribosomal RNA reads were removed. An average of 64.83% of filtered read counts were left after quality control filtering (Table S8).

Statistical analysis of 16S rRNA amplicon data and environmental variables

The rarefaction curves based on raw counts of the 16S rRNA gene amplicon data and diversity indices were calculated using the “vegan” (v.2.5-6) package (14) in R. Rarefaction curves of 16S rRNA gene sequences suggested that the majority of the microbial diversity had been covered (Fig. S3). Further analysis was carried out on samples with >500 counts.

Alpha Diversity (Shannon´s H) was normalized by scaling with ranked subsampling (15). A general linear model was used to perform an ANOVA test for significant differences between diversity indices between the two bays over the different sampling occasions. Due to the nature of the dataset, “bay” and sampling “month” with an interaction between both were selected as fixed variables while “replicate” was nested within the fixed variable “sampling site”. Different models were tested and the best fit was chosen based on the Akaike information criterion. A linear mixed model could not be chosen due to the small level size of the “sampling site” and “replicate”, even though it showed the best fit. An ANOVA with fixed variables and a good fit was chosen instead. The model was created with the “lm()” function within the “stats” (v. 4.0.4) package (3) in R and additional pairwise comparison of bays on each sampling month using the “emmeans” package (v. 1.5.4) was performed (Table S2) (16).

The mean and standard deviation were calculated for each environmental variable per bay, between the different sampling sites in each bay, and between the different sampling points over the year. A PCA analysis of the environmental variables of the different samples showed an influence of the season, bays, and sampling sites (Fig. S9). Therefore, statistical differences of tested environmental variables between bays was

carried out using the same model as used for diversity testing. The best fit was seen with an ANOVA with "bay" and "month" as fixed variables with involved interaction plus the nested and fixed variables "replicate" within "sampling site" ("lm()" function, "stats" package; Table S2). To compare the surface and bottom water temperature in each bay, a general linear model and ANOVA was used with fixed explanatory variables including "surface plus bottom water" and "month" as interaction factors along with "sampling site" (Table S2).

PerMANOVA with 999 permutations was used to test for statistical differences on bacterial communities comparing both bays, using the "adonis()" function from the "vegan" package (14) (Table S2). To account for the data structure, bay and month were considered as interactions while using the function "strata" to control for sampling site. The samples X1081, X1085, X1010, X1011, and X1012 (further details on the samples within Table S1) were excluded from the CCA, variance inflation factor (VIF) and Pearson correlation as those samples were missing geochemical analyses and therefore, could not increase the knowledge of interaction between microbial communities and environmental variables. CCA (n=67) based on relative abundances of ASVs with environmental parameters as explanatory variables was done with the vegan package in R. The CCA was chosen to have a direct gradient analysis method to analyze how much of the variation in the microbial community composition can be explained by environmental variables. To test for statistical significant environmental variables an ANOVA (n=999 permutations) was used. Permutations of the samples were performed within bays. The variance inflation factor (Table S2) based on the CCA (n=67) from the vegan package was used to determine if the measured environmental variables add new information to the differences between

the two groups. All measured variables were retained since all variables could explain part of the variation within the communities.

For differential abundance analysis on the bacterial community (*n*=72), taxa not seen in at least 20% of the samples were filtered out (removing rare abundant ASVs) and a zero-inflated negative binominal model was used to reduce zeros within the dataset. Differential abundance analysis was carried out on counts with "DESeq2" in R (v. 1.30.1) to calculated statistical differences between communities (Supplementary Table 4) (17). The model for the analysis included bay, month, and sampling site as fixed factors, while replicates were nested within the sampling sites, due to the influence of season and location on the bacterial communities.

Pearson correlation analysis was done comparing significant differential abundant ASVs over 0.5% relative abundance (*n*=67) summarized on family level for which counts were clr (centered log ratio) transformed using the packages "zComposition" (18) and "CoDaSeq" (19). Correlation of these data with environmental variables was then tested using the "ggcorrmat" function from the "ggstatsplot" package (20) with Benjamin-Hochberg correction for *p*-values (Table S5).

Meta-transcriptomic analysis

The rRNA filtered reads provided by JGI were used for the co-assembly of the metatranscriptomes. All samples were assembled using the program MEGAHIT (v. 1.2.9) using default settings (21). From each sample the sequence reads were mapped against the co-assembled contigs from the previous step using salmon (v. 1.1.0) with default settings (22). The raw counts were filtered with the contig threshold of at least five in at least three samples. In the next step, TPM (transcripts per million) values were calculated based on the counts of each contig and their length from the original co-assembly. The *de novo* assembly resulted in 1.2 million contigs of which 615327 (51.2%) could be annotated against the UniprotKB/SwissProt database (see below for methods). This resulted in a total of 3981 unique known genes with 3919 genes within the heated bay and 3939 genes in the control bay (Table S6). The filtered contigs were used for further analysis of functional annotation and differential expression analysis.

Meta-transcriptomic functional and taxonomical annotation

Functional annotation of the contigs was carried out by Prokka (v. 1.45) (23) with default settings annotating the bacteria kingdom using the supplied UniprotKB/SwissProt database (v. UniprotKB 2021_03; Table S6). Based on this data, additional functional annotation was done using eggNOG-mapper (emapper) on default settings (v. 2.0.8-2) retrieving additional information on KEGG, GO and PFAM identifiers based on the eggNOG database (v. eggNOG 5.0) (24). Taxonomic annotation of the contigs was performed using the default settings of the Contig Annotation Tool (v. 5.2.3) using the NR-database (25) (v. 2021-01-07; Table S6). For the calculation of the PCA (based on Euclidean distances), the functional TPM values were transformed using VST (variance-stabilizing transformation) within the DESeq2 package (v. 1.30.1) (17). Based on the TPM values and taxonomical annotation boxplots and bar plots were generated.

Meta-transcriptomic differential expression analysis

Differential expression analysis was performed using DESeq2 (v. 1.32.0) (17) on the filtered "raw" counts from the co-assembly. The two bays (n=12 per bay) were modeled as factors with the different sampling sites (n=3 per bay) as replicates to give differentially expressed genes over the year. Only significantly differential expressed genes were retained (adjusted p-value <0.05) and used for further analysis and visualization. A total of 30447 (2.5%, LFC >0) genes were significantly expressed within the warm bay while 24518 (2%, LFC <0) were significantly expressed within the control bay (Table S7). A volcano plot were plotted showing significant differential expressed genes (Fig. S10). The mean of the LFC values were calculated in each bay and used for Fig. 5. Since the DE

analysis was carried out by transcripts of genes, which could be from various sources (e.g. different organism), the overview of the DE genes in Fig. 5 could be also resulting in up regulations of both bays.

Support for the validity of the microbial communities within the bays

The heated bay has been used as a cooling outlet for the nearby nuclear power plant for at least 50 years. The intake water for the cooling system was from nearby open coastal water from 16-18 m below the sea surface (1) that is heated up to ~10 °C above the ambient water temperature within the system (1). Comparison of the 16S rRNA gene amplicon and RNA transcripts data suggested no noteworthy influence of the cooling water intake into the heated bay. For example, the dominant species annotated from the RNA transcripts in the heated bay could also be found within the control bay at lower numbers (e.g. *Thiobacillus*). Additionally, comparing data from benthic and open water communities within the Baltic Sea showed evidence that the microbes from in-flowing cooling water containing open Baltic Sea water did not noticeably influence the sediment microbial community composition (26-28).

References

1. Seidel L, Karjalainen M. Email from environmental engineer at the OKG to author. 08 June 2021.
2. Wickham H. ggplot2: Elegant Graphics for Data Analysis. New York: Springer-Verlag; 2016.
3. R Core Team. R: A language and environment for statistical computing. 2018.
4. Broman E, Li L, Fridlund J, Svensson F, Legrand C, Dopson M. Spring and late summer phytoplankton biomass impact on the coastal sediment microbial community structure. Microbial Ecology. 2018(77):288-303.
5. Heiri O, Lotter AF, Lemcke G. Loss on ignition as a method for estimating organic and carbonate content in sediments: reproducibility and comparability of results. Journal of Paleolimnology. 2001;25(1):101-10.
6. Dickens GR, Koelling M, Smith DC, Schnieders L, the Iodp Expedition Scientists. Rhizon sampling of pore waters on scientific drilling expeditions: an example from the IODP expedition 302, arctic coring expedition (ACEX). Scientific Drilling. 2007;4:22-5.
7. Mondol N, Bjørlykke K, Jahren J, Høeg K. Experimental mechanical compaction of clay mineral aggregate“ Changes in physical properties of mudstones during burial. Marine and Petroleum Geology. 2007;24:289-311.
8. Boudreau BP. The diffusive tortuosity of fine-grained unlithified sediments. Geochimica et Cosmochimica Acta. 1996;60(16):3139-42.
9. Hugerth LW, Wefer HA, Lundin S, Jakobsson HE, Lindberg M, Rodin S, et al. DegePrime, a program for degenerate primer design for broad-taxonomic-range PCR in microbial ecology studies. Applied Environmental Microbiology. 2014;80(16):5116-23.
10. Lindh MV, Figueroa D, Sjostedt J, Baltar F, Lundin D, Andersson A, et al. Transplant experiments uncover Baltic Sea basin-specific responses in bacterioplankton community composition and metabolic activities. Frontiers in Microbiology. 2015;6:223.
11. Reimann C, Filzmoser P, Fabian K, Hron K, Birke M, Demetriades A, et al. The concept of compositional data analysis in practice--total major element concentrations in agricultural and grazing land soils of Europe. Science of The Total Environment. 2012;426:196-210.
12. Aitchison J. The Statistical Analysis of Compositional Data. London: Chapman & Hall; 1986.
13. Bushnell B. BBMap. sourceforgenet/projects/bbmap/. 2014.
14. Oksanen J, Guillaume Blanchet F, Friendly M, Kindt R, Legendre P, McGlinn D, et al. vegan: community ecology package. 2.5-5 ed2019.
15. Beule L, Karlovsky P. Improved normalization of species count data in ecology by scaling with ranked subsampling (SRS): application to microbial communities. PeerJ. 2020;8:e9593.
16. Lenth RV. emmeans: estimated marginal means, aka least-squares means. 1.5.4 ed2021.
17. Love MI, Huber W, Anders S. Moderated estimation of fold change and dispersion for RNA-seq data with DESeq2. Genome Biology. 2014;15(12):550.
18. Palarea-Albaladejo J, Martín-Fernández JA. zCompositions — R package for multivariate imputation of left-censored data under a compositional approach. Chemometrics and Intelligent Laboratory Systems. 2015;143:85-96.

19. Gloor GB, Wu JR, Pawlowsky-Glahn V, Egozcue JJ. It's all relative: analyzing microbiome data as compositions. Annals of Epidemiology. 2016;26(5):322-9.
20. Patil I. ggstatsplot: 'ggplot2' based plots with statistical details. CRAN2018.
21. Culligan EP, Sleator RD. Editorial: from genes to species: novel insights from metagenomics. Frontiers in Microbiology. 2016;7:1181.
22. Patro R, Duggal G, Love MI, Irizarry RA, Kingsford C. Salmon provides fast and bias-aware quantification of transcript expression. Nature Methods. 2017;14(4):417-9.
23. Seemann T. Prokka: rapid prokaryotic genome annotation. Bioinformatics. 2014;30(14):2068-9.
24. Huerta-Cepas J, Szklarczyk D, Heller D, Hernández-Plaza A, Forslund SK, Cook H, et al. eggNOG 5.0: a hierarchical, functionally and phylogenetically annotated orthology resource based on 5090 organisms and 2502 viruses. Nucleic Acids Research. 2018;47(D1):D309-D14.
25. von Meijenfeldt FAB, Arkhipova K, Cambuy DD, Coutinho FH, Dutilh BE. Robust taxonomic classification of uncharted microbial sequences and bins with CAT and BAT. Genome Biology. 2019;20(1):217.
26. Klier J, Dellwig O, Leipe T, Jürgens K, Herlemann DPR. Benthic bacterial community composition in the oligohaline-marine transition of surface sediments in the Baltic Sea based on rRNA analysis. Frontiers in Microbiology. 2018;9(236).
27. Broman E, Sjostedt J, Pinhassi J, Dopson M. Shifts in coastal sediment oxygenation cause pronounced changes in microbial community composition and associated metabolism. Microbiome. 2017;5(1):96.
28. Broman E, Sachpazidou V, Pinhassi J, Dopson M. Oxygenation of hypoxic coastal Baltic Sea sediments impacts on chemistry, microbial community composition, and metabolism. Frontiers in Microbiology. 2017;8:2453.

Supplementary Figures

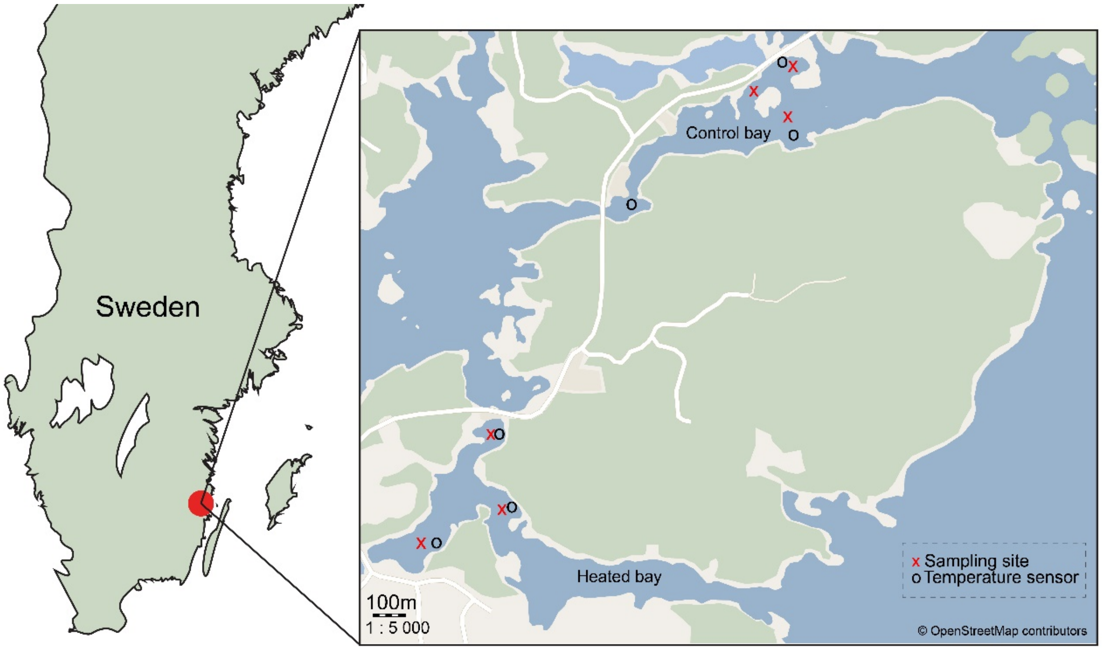

Fig. S1. Map of sampling area. Sampling area in southern Sweden, north of the city of Oskarshamn. The heated bay is in the south with the control bay in the north. There is no direct water connection between the two bays. Sampling sites in each bay are marked with red crosses while the locations of temperature sensors are marked with black circles.

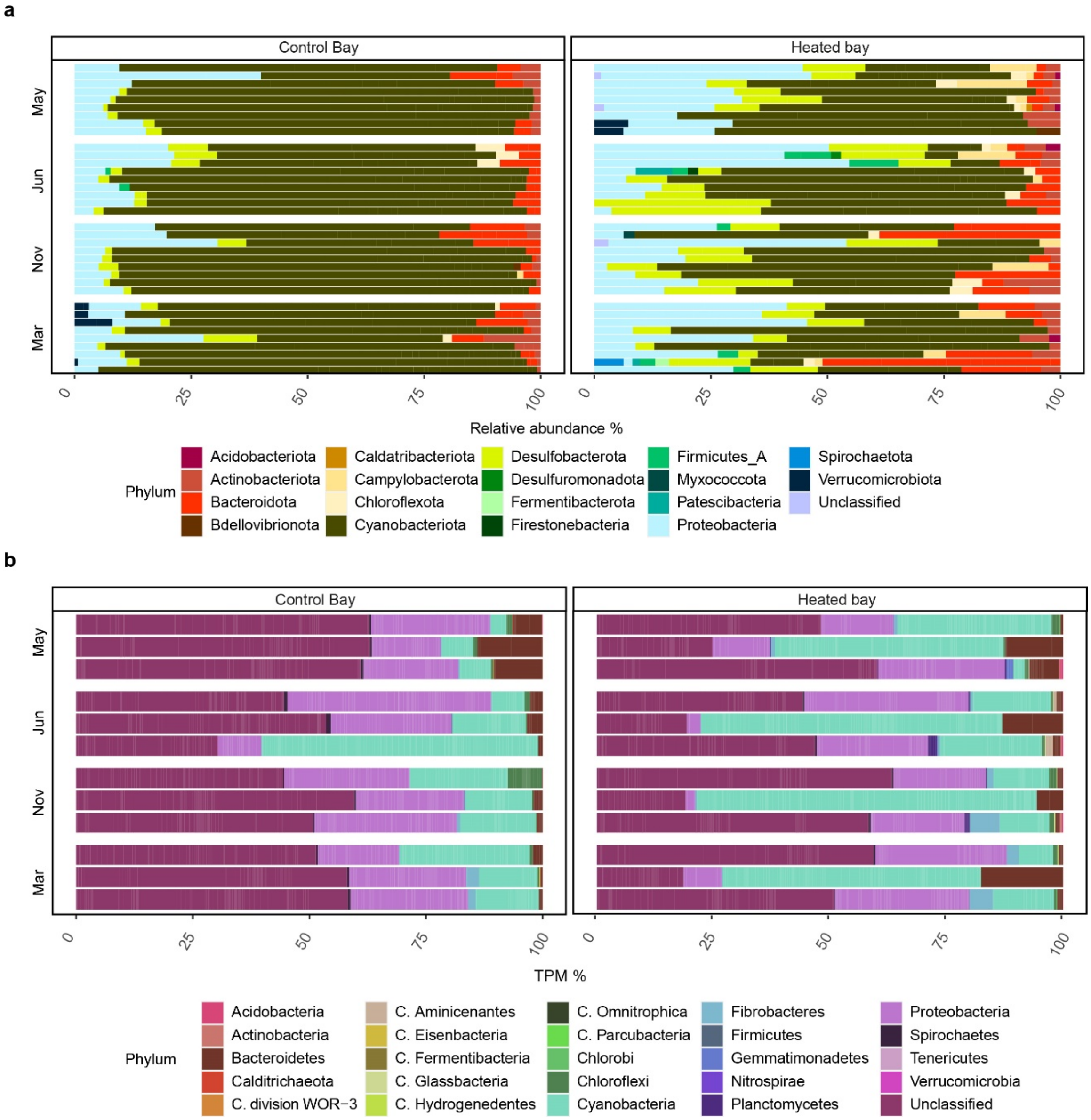

Fig. S2. 16S rRNA gene amplicon ASV on the phylum level. Taxonomic composition on phylum level of all ASVs over 0.5% relative abundance. Shown are a high relative abundance of Cyanobacteria in both bays but especially in the control bay. Most of the Cyanobacteria were unclassified on lower taxonomical levels (67% on family level) and have been filtered out for further analysis.

a

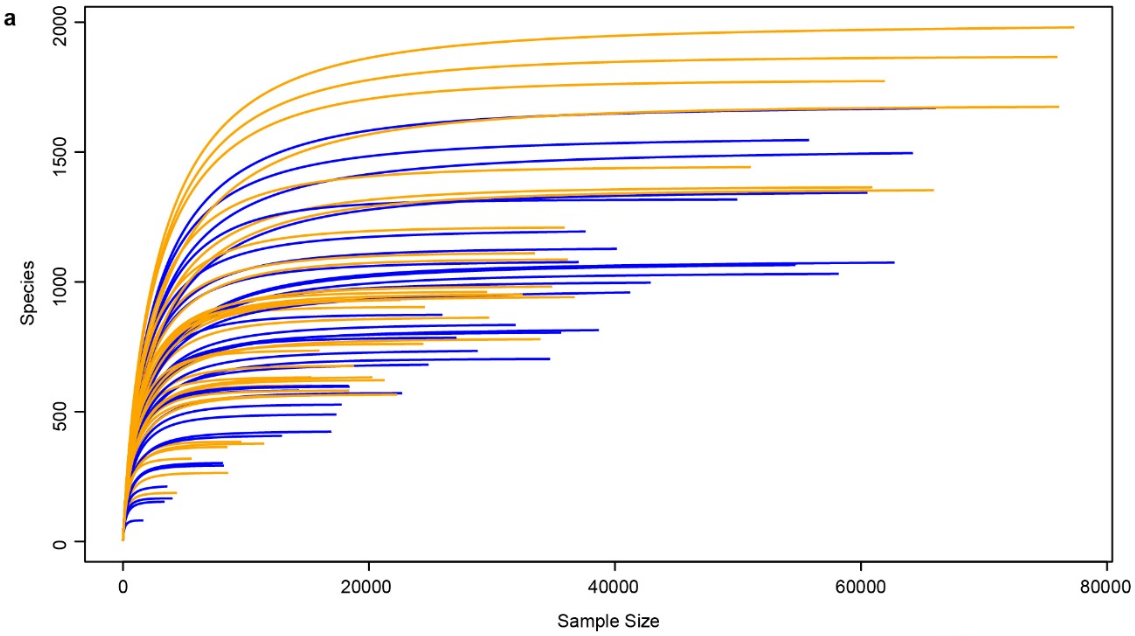

b

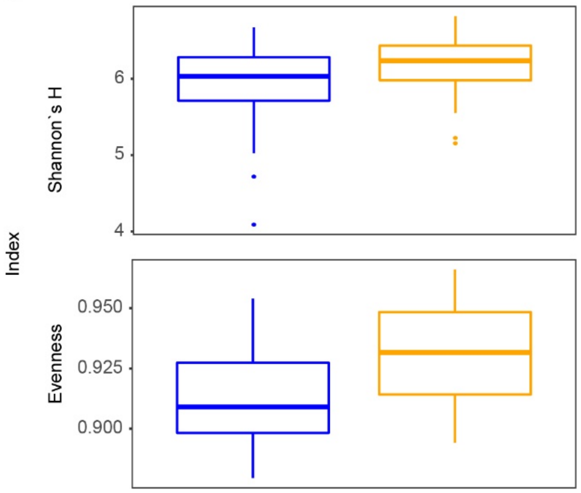

c

● Heated Bay
● Control Bay

Index	month	mean±s.d.
Shannon n = 9	May	● 6.05±0.91 ● 5.65±0.81
	Jun	● 6.00±0.47 ● 5.18±0.74
	Nov	● 6.54±0.36 ● 5.30±1.03
	Mar	● 6.29±0.41 ● 5.00±1.08
Evenness n = 9	May	● 0.87±0.04 ● 0.77±0.08
	Jun	● 0.86±0.05 ● 0.77±0.07
	Nov	● 0.89±0.03 ● 0.74±0.11
	Mar	● 0.88±0.04 ● 0.77±0.09

Fig. S3. 16S rRNA gene amplicon ASV data and diversity. (a) Sequencing depth of each sample showing good coverage of the diversity in each of the sites and (b) alpha diversity calculation on the dataset with singletons and doubletons filtered out (61 % single and doubletons) on each bay over the year underlines findings on the whole dataset. (c) Mean and standard deviation of the calculated diversity indices on the whole dataset (Shannon H & Shannon evenness) for each month (n=9) for the heated (orange) and control (blue) bays.

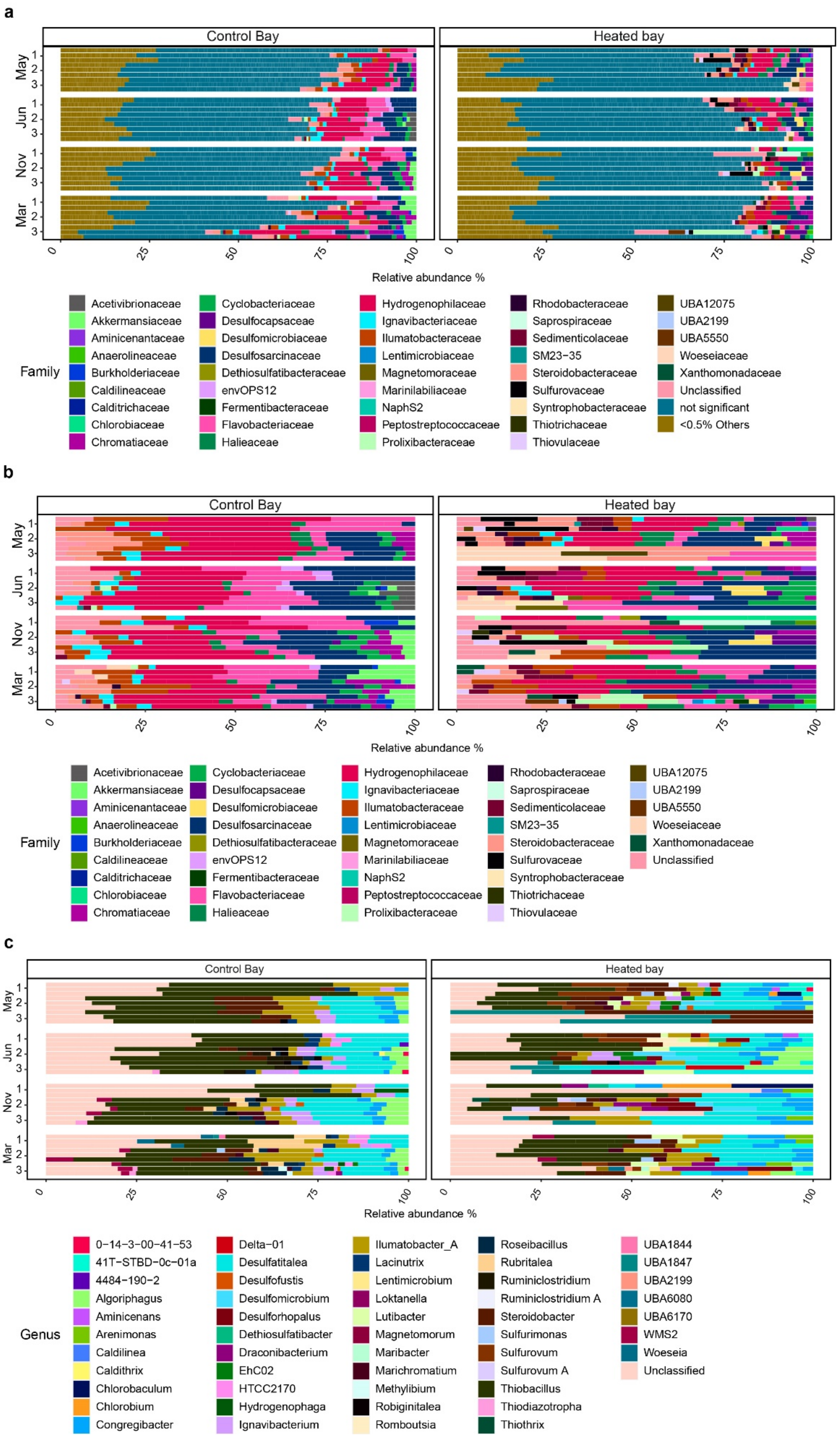
a
Control Bay
Heated bay
May
Jun
Nov
Mar
1
2
3
0
25
50
75
100
Relative abundance %
Family
Acetivibrionaceae
Akkermansiaceae
Aminicenantaceae
Anaerolineaceae
Burkholderiaceae
Caldilineaceae
Calditrichaceae
Chlorobiaceae
Chromatiaceae
Cyclobacteriaceae
Desulfocapsaceae
Desulfomicrobiaceae
Desulfosarcinaceae
Dethiosulfatibacteraceae
envOPS12
Fermentibacteraceae
Flavobacteriaceae
Halieaceae
Hydrogenophilaceae
Ignavibacteriaceae
Ilumatobacteraceae
Lentimicrobiaceae
Magnetomoraceae
Marinilabiliaceae
NaphS2
Peptostreptococcaceae
Prolixibacteraceae
Rhodobacteraceae
Saprospiraceae
Sedimenticolaceae
SM23−35
Steroidobacteraceae
Sulfurovaceae
Syntrophobacteraceae
Thiotrichaceae
Thiovulaceae
UBA12075
UBA2199
UBA5550
Woeseiaceae
Xanthomonadaceae
Unclassified
not significant
<0.5% Others
b
Control Bay
Heated bay
May
Jun
Nov
Mar
1
2
3
0
25
50
75
100
Relative abundance %
Family
Acetivibrionaceae
Akkermansiaceae
Aminicenantaceae
Anaerolineaceae
Burkholderiaceae
Caldilineaceae
Calditrichaceae
Chlorobiaceae
Chromatiaceae
Cyclobacteriaceae
Desulfocapsaceae
Desulfomicrobiaceae
Desulfosarcinaceae
Dethiosulfatibacteraceae
envOPS12
Fermentibacteraceae
Flavobacteriaceae
Halieaceae
Hydrogenophilaceae
Ignavibacteriaceae
Ilumatobacteraceae
Lentimicrobiaceae
Magnetomoraceae
Marinilabiliaceae
NaphS2
Peptostreptococcaceae
Prolixibacteraceae
Rhodobacteraceae
Saprospiraceae
Sedimenticolaceae
SM23−35
Steroidobacteraceae
Sulfurovaceae
Syntrophobacteraceae
Thiotrichaceae
Thiovulaceae
UBA12075
UBA2199
UBA5550
Woeseiaceae
Xanthomonadaceae
Unclassified
c
Control Bay
Heated bay
May
Jun
Nov
Mar
1
2
3
0
25
50
75
100
Relative abundance %
Genus
0−14−3−00−41−53
41T−STBD−0c−01a
4484−190−2
Algoriphagus
Aminicenans
Arenimonas
Caldilinea
Caldithrix
Chlorobaculum
Chlorobium
Congregibacter
Delta−01
Desulfatitalea
Desulfofustis
Desulfomicrobium
Desulforhopalus
Dethiosulfatibacter
Draconibacterium
EhC02
HTCC2170
Hydrogenophaga
Ignavibacterium
Ilumatobacter_A
Lacinutrix
Lentimicrobium
Loktanella
Lutibacter
Magnetomorum
Maribacter
Marichromatium
Methylibium
Robiginitalea
Romboutsia
Roseibacillus
Rubritalea
Ruminiclostridium
Ruminiclostridium A
Steroidobacter
Sulfurimonas
Sulfurovum
Sulfurovum A
Thiobacillus
Thiodiazotropha
Thiothrix
UBA1844
UBA1847
UBA2199
UBA6080
UBA6170
WMS2
Woeseia
Unclassified

Fig. S4. Seasonal influence on significantly differential abundant ASVs on family and genus level in the two bays. Differential abundance analysis on ASVs. (a) Significant differently abundant ASVs over 0.5% relative abundance summarized on family level as well as insignificant taxa in blue, and taxa under 0.5% relative abundance shown in dark yellow. (b) Significant ($p<0.05$) differently abundant ASVs over 0.5% (phylum Cyanobacteria filtered out) relative abundance summarized on family level in each bay and sampling time. (c) Significant differently abundant ASVs over 0.5% relative abundance summarized on genus Level.

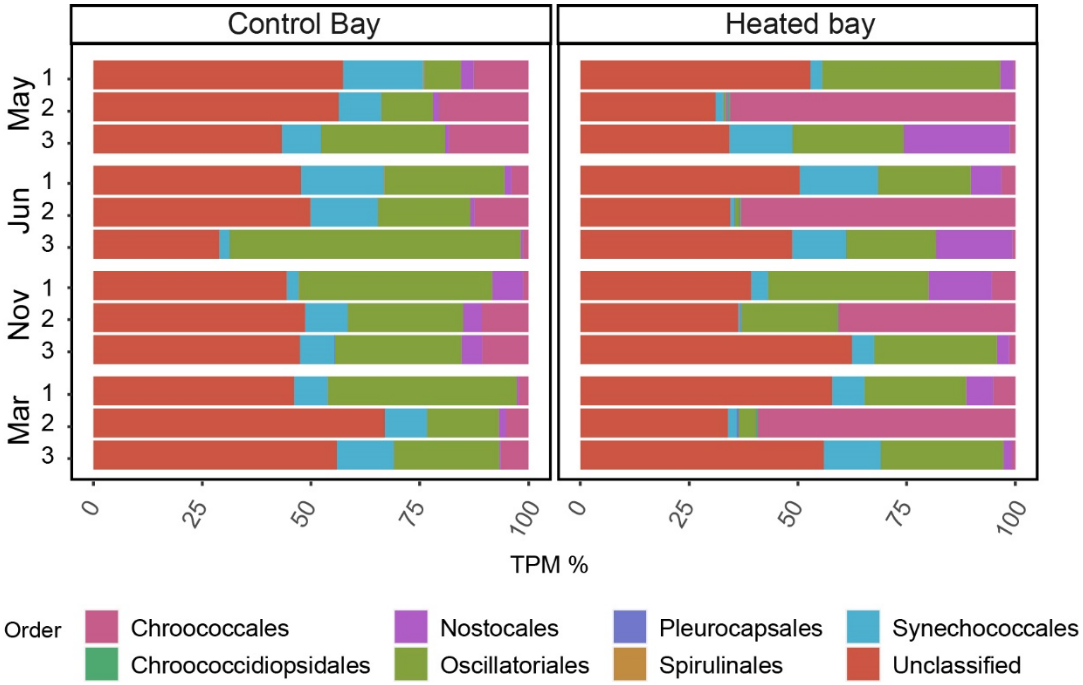

Fig. S5. RNA transcript based activity of Cyanobacteria orders. TPM of Cyanobacteria orders for the different sampling times and replicate sampling sites.

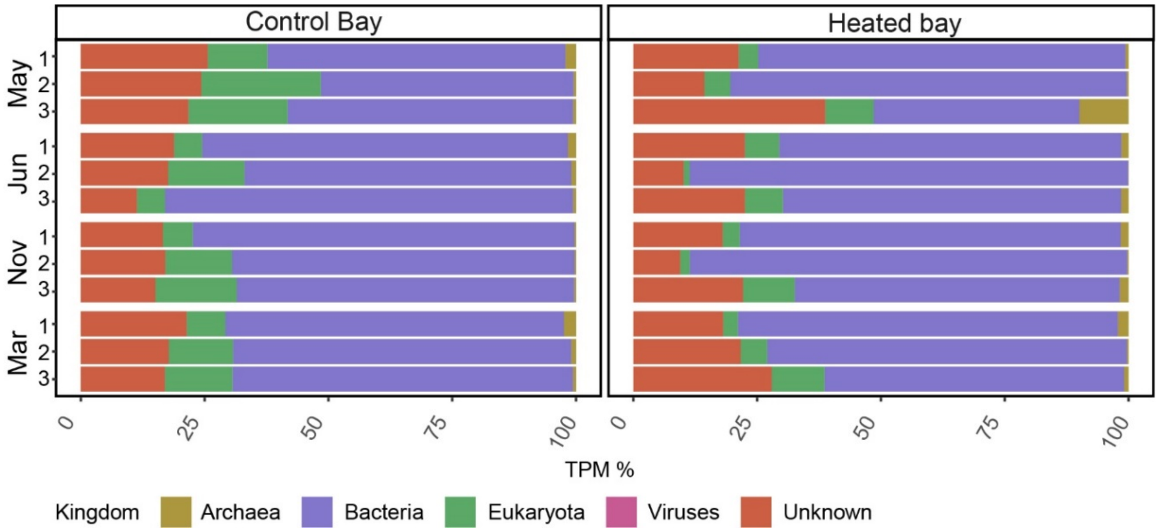

Fig. S6. Annotated kingdoms of RNA transcripts. Annotated taxa of the meta-transcriptomic dataset in the heated and control bay showing TPMs Archaea, Bacteria, Eukaryota kingdoms plus viruses for each sampling site (*n*=3) within each bay and sampling time (*n*=4).

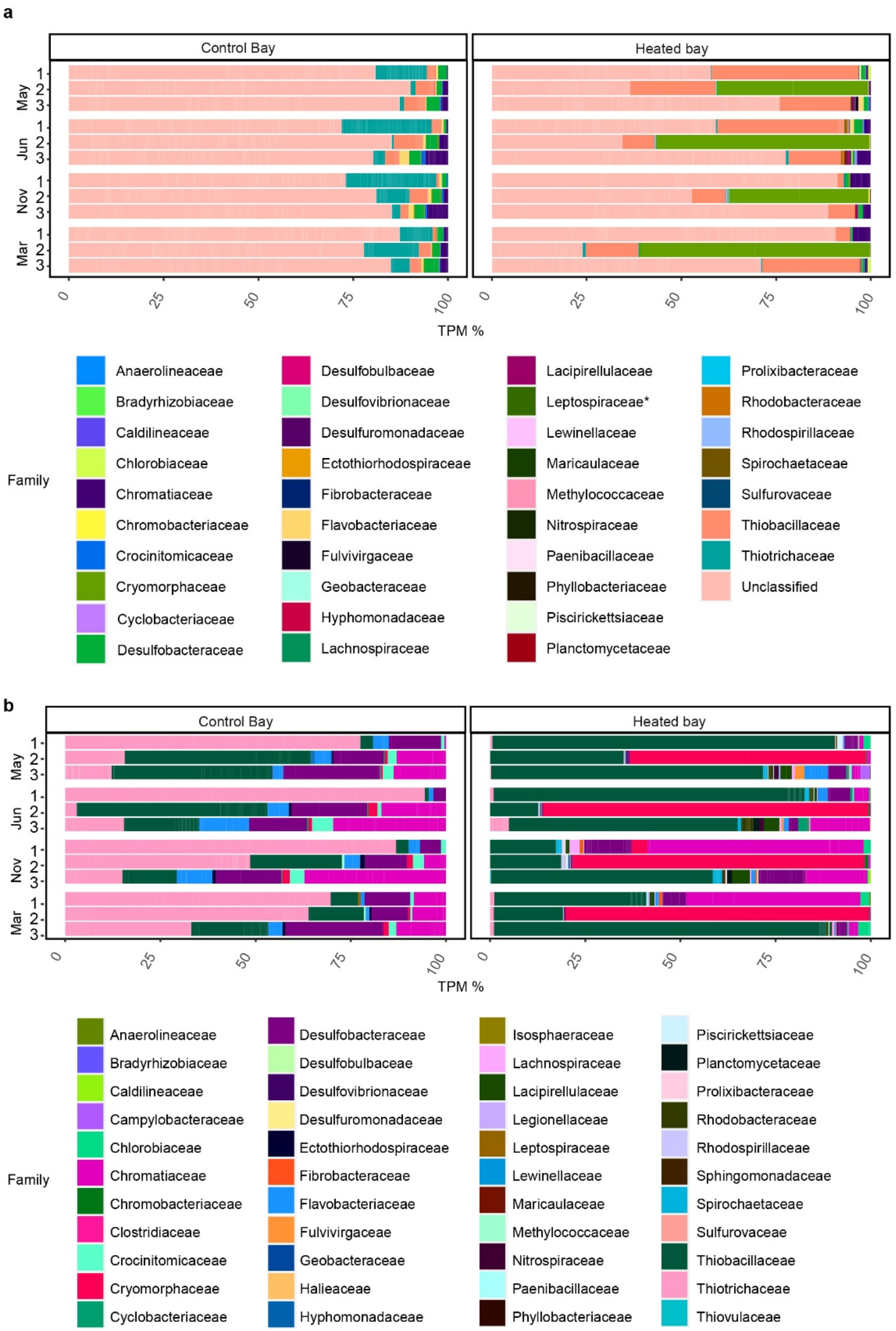

Fig. S7. Taxa associated with significantly different RNA transcripts. Different RNA transcripts with adjusted *p*-value <0.05 associated with taxa on the family level in the two bays. (a) Top 500 TPM values per sample in % for the sampling times (*n*=4) and locations (*n*=3). (b) Top 500 TPM values per sample of significantly different bacterial RNA transcripts (*p*-value adjusted <0.05) after removal of Unclassified for the sampling times (*n*=4) and locations (*n*=3).

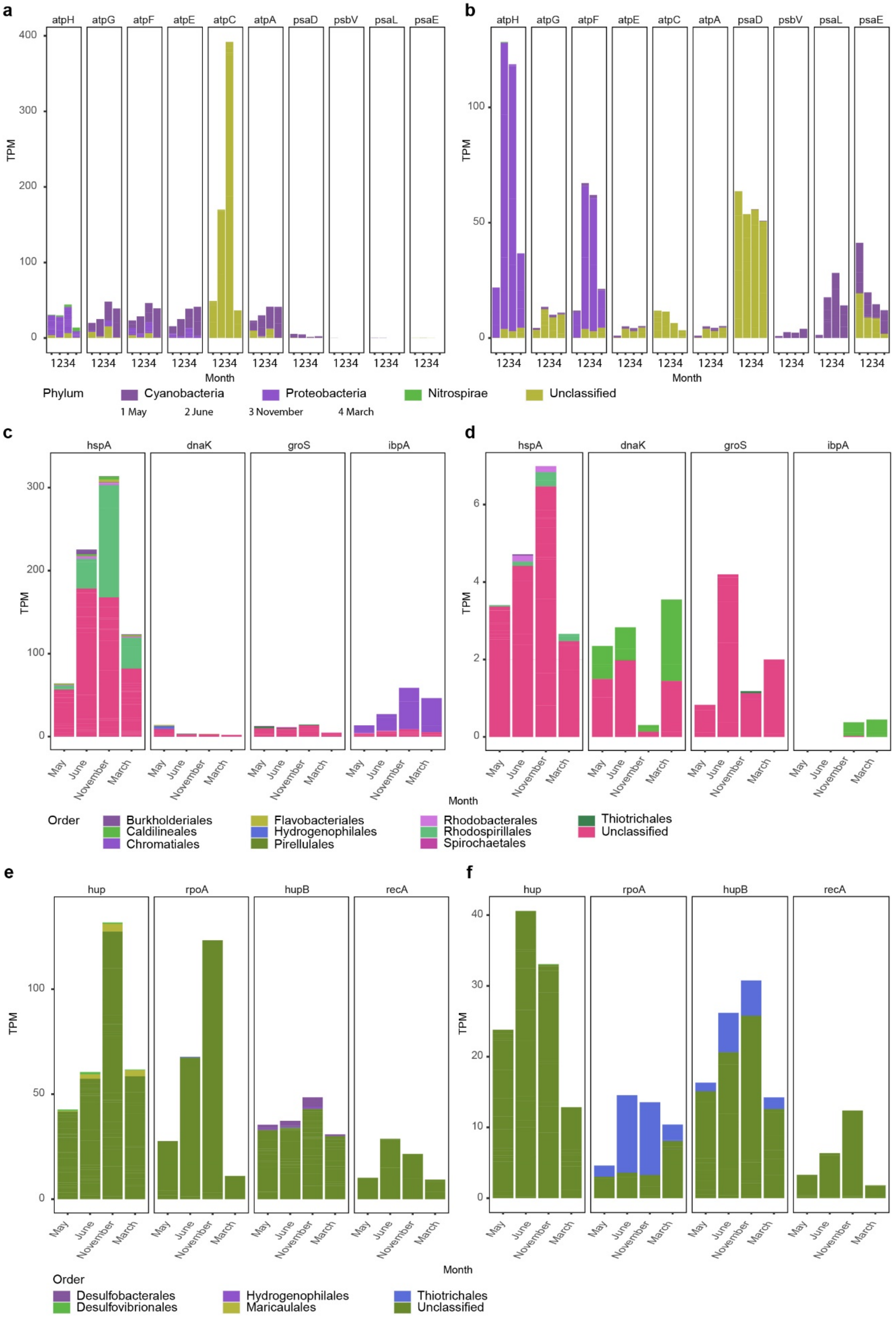

a
b
atpH atpG atpF atpE atpC atpA psaD psbV psaL psaE
TPM
Month
Phylum
Cyanobacteria
Proteobacteria
Nitrospirae
Unclassified
1 May
2 June
3 November
4 March
c
d
hspA dnaK groS ibpA
May June November March
Order
Burkholderiales
Caldilineales
Chromatiales
Flavobacteriales
Hydrogenophilales
Pirellulales
Rhodobacterales
Rhodospirillales
Spirochaetales
Thiotrichales
Unclassified
e
f
hup rpoA hupB recA
Order
Desulfobacterales
Desulfovibrionales
Hydrogenophilales
Maricaulales
Thiotrichales
Unclassified

Fig. S8. Overview of significant differential expressed genes LFC >5 related to photosynthesis, electron transport, stress and repair. Genes within the photosynthesis and photosynthesis related gene categories with a LFC >5 in at least one sample within either the heated or control bay. Shown are the TPM values (y-axis) of the genes (top) during the different sampling months (1=May, 2=June, 3=November, and 4=March) and their associated taxa on phylum level within the heated (a) and the control (b) bays. The y-axis is adjusted between the two graphs for better visualization of both bays (TPM 0-400 heated bay and 0-150 control bay); Genes within the chaperone category with a LFC >5 in at least one sample within either the heated or control bay. Shown are the TPM values (y-axis) of the genes (top) during the different sampling month and their associated taxa on phylum level within the heated (c) and the control (d) bays. The y-axis is adjusted between the two graphs for better visualization of both bays (TPM 0-300 heated bay and 0-10 control bay); Genes within the repair and recombination category with a LFC >5 in at least one sample within either the heated or control bay. Shown are the TPM values (y-axis) of the genes (top) during the different sampling month and their associated taxa on phylum level within the heated (e) and the control (f) bays. The y-axis is adjusted between the two graphs for better visualization of both bays (TPM 0-150 heated bay and 0-40 control bay).

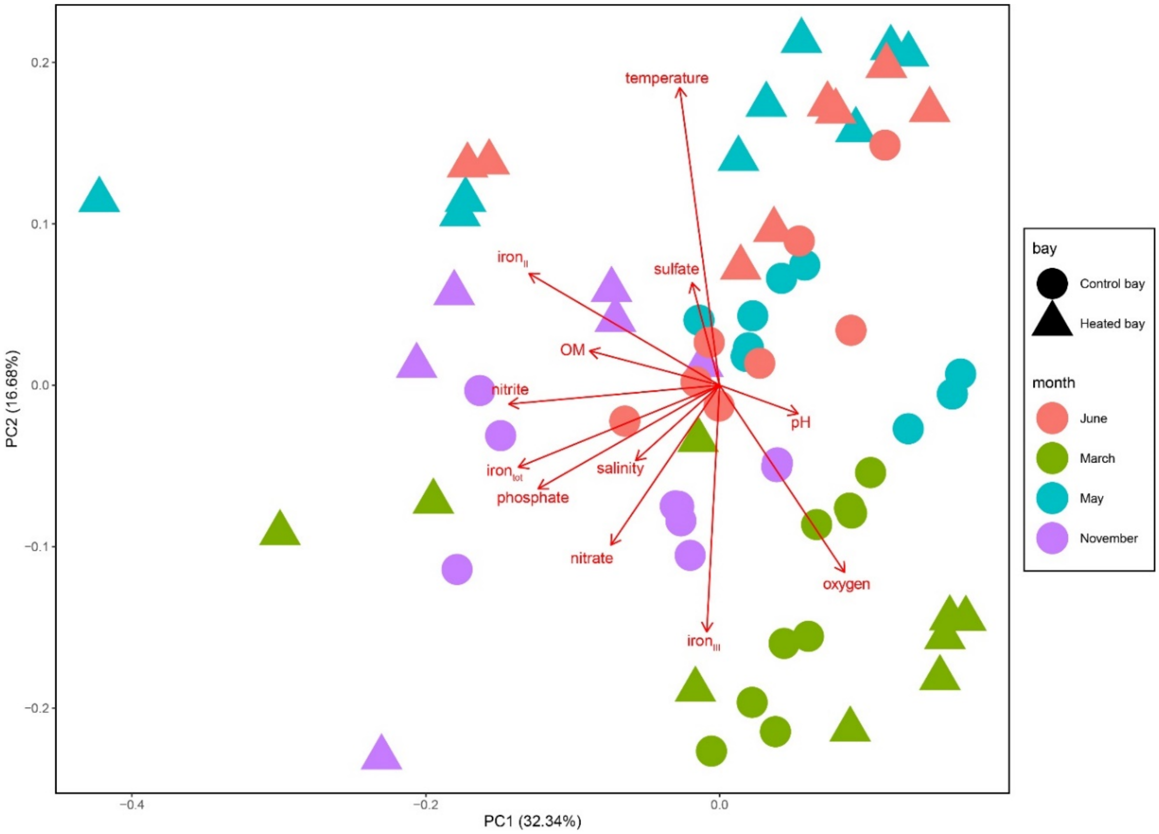

Fig. S9. Principal component analysis on environmental variables. Principal component analysis (PCA) on environmental variables of samples (*n*=67) taken at all sampling times in the two bays. A higher seasonal influence compared to locational differences was observed, therefore bay and season are influencing factors, which have been used as fixed effects within statistical modeling.

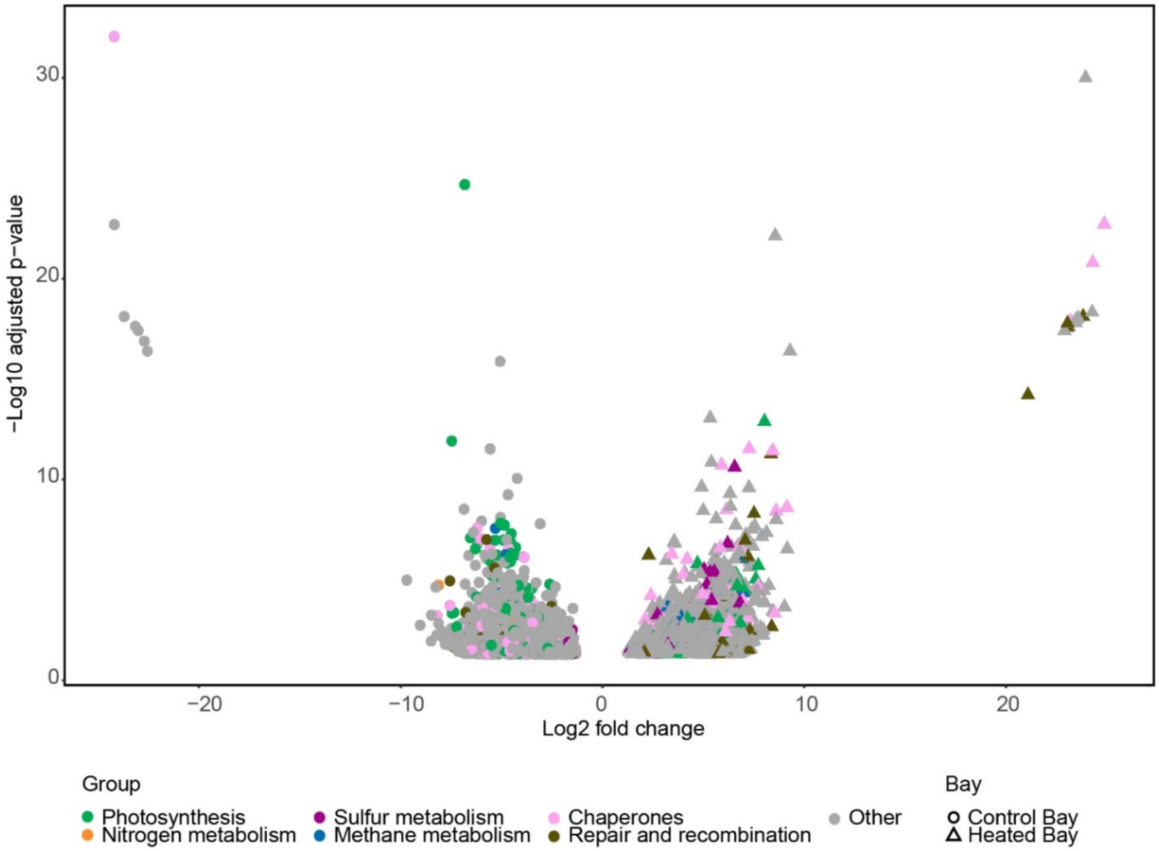

Fig. S10. Volcano plot of significantly different RNA transcripts between bays. Volcano plot of *p*-value adjusted significantly different RNA transcripts (%) in the heated (▲) and control (●) bays. Shown are the $\log_2$ fold change on the x-axis against the $-\log_{10}$ adjusted *p*-value on the y-axis. The genes are colored according to different selected groups based on KEGG divisions while the remaining genes were summarized as "others".

Supplementary Tables Information

Table S1. Sample information. Metadata table for each sample including sampling times, locations, and environmental variables.

Table S2. Statistical tests. Comparison of bays community composition, diversity indices, and environmental variables using different statistical approaches.

Table S3. Annotation of 16S rRNA amplicon sequence variance (ASV). Table of annotated taxonomy of the 16S rRNA gene amplicon sequences counts in each sample. A total of 20340 ASVs could be detected of which 61 % were single- or doubletons (12604).

Table S4. Differential abundance analysis of 16S rRNA gene amplicon data. Differential abundance analysis on ASVs for each month comparing bays using the contrast function of DESeq2; ASV = amplicon sequence variant number, baseMEAN, mean Control bay, mean Warm bay; log2FoldChange; lfcSE= LogFoldChange Standard Error; stats; *p*-value; padj = adjusted *p*-value with Benjamin Hochberg correction; Kingdom, Phylum, Class, Order, Family, Genus, Species.

Table S5. Pearson correlation of significant families on environmental variables. Pearson correlation analysis of significant taxa on family level and environmental variables. Each taxa was tested against each environmental variable in all samples (n=67). Listed are family taxa, environmental variable, estimate, confidence level, confidence low, confidence high, statistic, degrees of freedom error, Benjamin-Hochberg corrected *p*-value, method, and number of samples. Positive estimates show a positive correlation between two variables while negative estimates indicate a negative correlation.

Table S6. Functional & taxonomical annotation of known RNA transcripts in Bacteria & Archaea. Overview about the known functional plus taxonomical annotation based on Prokka and EGGnog plus TPM value of each sample within the kingdoms Bacteria and Archaea. Listed are information about the gene name (based on EGGnog) and best description of the gene (Best og description); the functional annotation using the GO-, EC-, KEGG and PFAM identification number; as well as the taxonomical annotation on Kingdom, Phylum, Class, Order, Family, Genus and Species levels. The table can be found on https://github.com/laseab/CC_WR.

Table S7. Significantly different RNA transcripts. Overview about adjusted *p*-value for significant differential RNA transcripts between the heated and control bay. (a) Shown are the baseMean, log2FoldChange (positive value shows higher transcript number in the heated bay compared to control bay while negative value shows higher transcript number in the control bay compared to the heated bay), standard error, statistic, *p*-value, adjusted *p*-value, COG ID, gene name, product and annotated taxonomy. (b) Shown are known differential expressed genes, with additional information to (a) with hierarchical groupings based on KEGG and the assigned groups from the different used figures.

Table S8. Sequencing information. (a) Sequencing and quality control of 16S rRNA gene amplicon and RNA for meta-transcriptomic analysis. Included are information on 16S rRNA gene amplicon sequencing with read length during sequencing, read length in million reads per sample, amount of sequences introduced in the dada2 pipeline before and after quality trimming and filter step, as well as the number of ASVs. (b) Information about meta-transcriptome sequencing include read length during sequencing, read count, filtered read count, and the percentage of read pairs merged.

Supplementary Table 2

Statistical approaches

1) Comparison of heated bay and control bay community composition with perMANOVA using the adonis function in the vegan package. The structure of the data were considered with bay and month as interaction, while using the function strata to control for sampling site.

	F-Model	R2	p-value	Permutations
Bay	13.99	0.1565	0.001***	999
Month	2.06	0.0693	0.001***	999
Bay:month	1.73	0.0581	0.001***	999

***: p≤0.001

2) Comparison of surface and bottom water temperature in each bay using a general linear model with lm and anova function. The model used fixed explanatory variables, including surface plus bottom water and month as interaction factors, as well as sampling site.

Bay	Variable	Df	SumSquares	MeanSquares	F	P-value
Heated Bay	Surface & bottom water	1	0	0	0.01	0.929
	Month	3	1283.33	427.78	683.95	<0.0001***
	Samplingsite	2	6.44	3.22	5.15	0.008**
	Surface & bottom water:month	3	3.75	1.25	1.99	0.123
Control Bay	Surface & bottom water	1	23.80	23.80	27.03	<0.0001***
	Month	3	1086.81	362.27	411.46	<0.0001***
	Samplingsite	2	50.45	25.23	28.65	<0.0001***
	Surface & bottom water:month	3	53.59	17.99	20.43	<0.0001***

***: p<0.001 **: p<0.01 *: p<0.05

1) A general linear model using the lm and anova function to test differences of environmental variables between bays, month & sampling sites (a). The model used fixed explanatory variables, including bay and month as interaction factors and replicates nested within sampling sites. Function emmeans was used to do pairwise comparison between bays on every sampling month (b); Df = degrees of freedom, Sum Sq = Sum of Squares, Mean Sq = mean of squares, SE = standard error.

(a)

Env.var	Variable	Df	Sum Sq.	Mean Sq.	F-value	P-value
Oxygen	Bay	1	22.01	22.01	9.84	2.87^{-03**}
(Bottom Water)	Month	3	126.87	42.29	18.91	2.76^{-08***}
	Samplingsite	4	48.20	12.05	5.39	1.11^{-03***}
	Bay:Month	3	9.63	3.21	1.43	0.24
	Samplingsite: Replicate	6	0.79	0.13	0.05	0.99
Temperature	Bay	1	568.40	568.40	735.54	2.2^{-16***}
(Bottom Water)	Month	3	972.99	324.33	419.70	2.2^{-16***}
	Samplingsite	4	42.94	10.73	13.89	1.20^{-07***}
	Bay:Month	3	43.09	14.36	18.58	3.46^{-08***}
	Samplingsite: Replicate	6	0.16	0.03	0.03	0.99
Salinity	Bay	1	0.01	0.01	137.44	7.93^{-16***}
(bottom water)	Month	3	0.03	0.01	145.73	2.20^{-16***}
	Samplingsite	4	1.11^{-03}	4.43^{-03}	5.03	1.77^{-02**}
	Bay:Month	3	0.01	0.01	56.97	5.30^{-16***}
	Samplingsite: Replicate	6	9.00^{-05}	1.50^{-05}	0.02	0.99
pH	Bay	1	6.20^{-02}	6.21^{-02}	0.10	0.74
	Month	3	6.13	2.04	34.69	3.56^{-12***}
	Samplingsite	4	1.74	0.43	7.38	9.78^{-05***}
	Bay:Month	3	0.13	0.04	0.79	0.50
	Samplingsite: Replicate	6	0.07	0.01	0.22	0.96
Sulfate	Bay	1	0.12	0.12	13.95	4.88^{-03***}
	Month	3	2.34	0.78	89.84	2.2^{-16***}
	Samplingsite	4	0.27	0.06	7.83	5.76^{-05***}

	Bay:Month	3	0.11	0.03	4.48	7.365^{-02**}
	Samplingsite: Replicate	6	0.04	6.79^{-02}	0.77	0.59
Total Iron	Bay	1	0.88	0.88	0.27	0.60
	Month	3	24.90	8.30	2.59	0.06
	Samplingsite	4	153.03	38.25	11.95	7.43^{-07***}
	Bay:Month	3	21.14	7.04	2.20	0.09
	Samplingsite: Replicate	6	10.82	1.80	0.56	0.75
Ferrous Iron	Bay	1	2.98	2.98	1.24	0.27
	Month	3	34.33	11.44	4.75	5.44^{-2**}
	Samplingsite	4	192.38	48.09	19.99	8.12^{-10***}
	Bay:Month	3	18.27	6.09	2.53	0.06
	Samplingsite: Replicate	6	3.91	0.65	0.27	0.94
Nitrate	Bay	1	0.29	0.29	4.96	0.03^{*}
	Month	3	0.34	0.11	1.96	0.13
	Samplingsite	4	1.10	0.27	4.71	2.68^{-02**}
	Bay:Month	3	0.10	0.03	0.60	0.61
	Samplingsite: Replicate	6	0.08	0.01	0.23	0.96
Nitrite	Bay	1	1.00^{-03}	1.30^{-03}	2.00^{-02}	0.96
	Month	3	0.97	0.32	5.05	4.50^{-02**}
	Samplingsite	4	4.98	1.24	19.25	1.42^{-09***}
	Bay:Month	3	0.36	0.12	1.86	0.14
	Samplingsite: Replicate	6	0.14	0.02	0.37	0.89
Phosphate	Bay	1	0.15	0.15	3.05	0.08
	Month	3	1.35	0.45	8.79	9.05^{-05***}
	Samplingsite	4	3.49	0.87	16.98	8.43^{-09***}
	Bay:Month	3	0.21	0.07	1.41	0.24
	Samplingsite: Replicate	6	0.24	0.04	0.78	0.58
Organic matter	Bay	1	516.15	516.15	11.39	1.44^{-02***}

Month	3	150.41	50.14	1.10	0.35
Samplingsite	4	437.81	109.45	2.41	0.06
Bay:Month	3	942.38	314.13	6.93	5.54^{-03***}
Samplingsite: Replicate	6	410.08	68.35	1.50	0.19

***: $p<0.001$ **: $p<0.01$ *: $p<0.05$

(b)

**Env.var**	**month**	**estimate**	**SE**	**Df**	**t.ratio**	**p-value**
oxygen	June	1.425	0.758	49	1.879	0.066
(bottom water)	March	0.413	0.705	49	0.586	0.560
	May	2.453	0.705	49	3.480	0.001***
	November	1.107	0.815	49	1.359	0.180
Temperature	June	-7.940	0.446	49	-17.809	<0.0001***
(bottom water)	March	-5.370	0.414	49	-12.951	<0.0001***
	May	-6.300	0.414	49	-15.203	<0.0001***
	November	-3.170	0.479	49	-6.622	<0.0001***
Salinity	June	-0.015	0.004	49	-3.151	0.002**
(bottom water)	March	-0.028	0.004	49	-6.424	<0.0001***
	May	-0.002	0.004	49	-0.515	0.608
	November	-0.086	0.005	49	-16.926	<0.0001***
pH	June	-0.078	0.123	49	-0.640	0.525
	March	0.130	0.114	49	1.139	0.260
	May	-0.021	0.114	49	-0.185	0.853
	November	0.145	0.132	49	1.099	0.277
Sulfate	June	0.006	0.047	49	0.139	0.889
	March	0.094	0.043	49	2.157	0.035
	May	0.025	0.043	49	0.591	0.557
	November	0.244	0.050	49	4.823	<0.0001***
Total iron	June	-1.132	0.907	49	-1.248	0.218
	March	-0.732	0.843	49	-0.868	0.389

	May	-0.962	0.843	49	-1.141	0.259
	November	1.939	0.975	49	1.99	0.052
Ferrous Iron	June	-1.358	0.786	49	-1.726	0.090
	March	1.142	0.731	49	1.562	0.124
	May	-1.341	0.731	49	-1.835	0.072
	November	0.128	0.845	49	0.152	0.879
Nitrate	June	0.060	0.123	49	0.488	0.627
	March	0.250	0.114	49	2.196	0.032*
	May	0.163	0.114	49	1.426	0.160
	November	0.057	0.132	49	0.437	0.664
Nitrite	June	0.175	0.129	49	1.362	0.179
	March	0.026	0.120	49	0.221	0.825
	May	-0.210	0.120	49	-1.759	0.084
	November	0.049	0.139	49	0.354	0.724
Phosphate	June	0.110	0.115	49	0.963	0.340
	March	-0.084	0.107	49	-0.788	0.434
	May	0.188	0.107	49	1.762	0.084
	November	0.196	0.124	49	1.590	0.118
Organic matter	June	1.360	3.410	49	0.397	0.692
	March	-1.650	3.170	49	-0.520	0.605
	May	-17.590	3.170	49	-5.545	<0.0001***
	November	-3.270	3.670	49	-0.892	0.376

***: p<0.001 **: p<0.01 *: p<0.05

2) Variance inflation factor (VIF) for CCA to test if environmental variables add explanatory information to the differences between communities. VIF < 5 indicate variables still explain part of the differences between bays communities.

Environmental variable	VIF
Temperature	2.700747
Oxygen	2.512254
Phosphate	3.553445
Total Iron	4.104953
Ferrous Iron	3.205628
pH	3.397552
Sulfate	2.126604
Nitrate	1.659459
Nitrite	4.993285
Organic matter	1.372783
Salinity	2.219296
Depth	3.128763

3) Permutation test for CCA under direct model with marginal effects of terms within each bay. Number of permutations *n*= 999. Test of which environmental variables significantly explain variation on microbial communities in each bay.

Variables	Df	ChiSquare	F	p-value
Total iron	1	0.130	1.273	0.043*
pH	1	0.125	1.223	0.067
Phosphate	1	0.174	1.695	0.001***
Sulfate	1	0.128	1.249	0.044*
Nitrate	1	0.183	1.788	0.007**
Nitrite	1	0.143	1.396	0.156
Ferrous Iron	1	0.189	1.849	0.007**
Temperature	1	0.202	1.975	0.004**
Oxygen	1	0.142	1.385	0.006**
OM	1	0.092	0.901	0.822
Salinity	1	0.205	2.001	0.021*
Depth	1	0.193	1.882	0.001***

***: $p<0.001$ **: $p<0.01$ *: $p<0.05$

4) A general linear model using the lm and anova function to test differences of diversity indices (Shannon H & Shannon evenness) between bays and month (a). The model used fixed explanatory variables, including bay and month as interaction factors and replicates nested within sampling sites. Function emmeans was used to do pairwise comparison between bays on every sampling month (b). Df = degrees of freedom, Sum Sq = Sum of Squares, Mean Sq = Mean of Squares, SE = standard error.

(a)

**Diversity Index**	**Variable**	**Df**	**Sum Sq.**	**Mean Sq.**	**F-value**	**P-value**
Shannon H	Bay	1	15.80	15.80	59.78	2.67^{-10***}
	Month	3	1.38	0.46	1.74	0.17
	Samplingsite	4	23.78	5.95	22.50	5.67^{-11***}
	Bay:Month	3	2.30	0.77	2.90	0.04^{*}
	Samplingsite: Replicate	6	0.57	0.10	0.36	0.90
Evenness	Bay	1	0.23	0.23	234.93	2.00^{-16***}
	Month	3	1.12^{-3}	3.96^{-3}	0.40	0.75
	Samplingsite	4	0.30	7.51^{-2}	76.40	2.00^{-16***}
	Bay:Month	3	7.56^{-3}	2.56	2.56	0.06
	Samplingsite: Replicate	6	1.04^{-3}	0.18	0.18	0.98

***: p≤0.001 **: p≤0.01 *: p≤0.05

(b)

**Diversity Index**	**variable**	**month**	**estimate**	**SE**	**Df**	**t.ratio**	**p-value**
Shannon H	Bay	May	-0.818	0.242	54	-3.373	0.0014^{***}
		June	-1.289	0.242	54	-5.318	$<0.0001^{***}$
		November	-0.405	0.242	54	-1.670	0.1006
		March	-1.237	0.242	54	-5.102	$<0.0001^{***}$
Evenness	Bay	May	-0.093	0.014	54	-6.321	$<0.0001^{***}$
		June	-0.112	0.014	54	-7.579	$<0.0001^{***}$
		November	-1.008	0.014	54	-6.819	$<0.0001^{***}$
		March	-0.146	0.014	54	-9.936	$<0.0001^{***}$

***: p≤0.001 **: p≤0.01 *: p≤0.05

5) A general linear model using the lm and anova function to test differences of diversity indices (Shannon H & Shannon evenness) on the doubleton removed data between bays and month (a). The model used fixed explanatory variables, including bay and month as interaction factors and replicates nested within sampling sites.

**Diversity Index**	**Variable**	**Df**	**Sum Sq.**	**Mean Sq.**	**F-value**	**P-value**
Shannon H	Bay	1	1.57	1.57	10.01	0.0025**
	Month	3	1.92	0.64	4.07	0.0111*
	Samplingsite	4	4.07	1.01	6.46	0.0002***
	Bay:Month	3	2.67	0.89	5.64	0.0019**
	Samplingsite: Replicate	6	0.37	0.06	0.39	0.8793
Evenness	Bay	1	0.0066	0.0066	40.48	<0.0001***
	Month	3	0.0010	0.0003	2.09	0.1113
	Samplingsite	4	0.0162	0.0040	24.86	<0.0001***
	Bay:Month	3	0.0019	0.0006	3.95	0.0127*
	Samplingsite: Replicate	6	0.0002	0.0001	0.23	0.9614

***: p≤0.001 **: p≤0.01 *: p≤0.05
