# Supplementary material for: Weakened resilience of benthic microbial communities in the face of climate change
Source: ISME Commun. 2022 Mar 8;2:21. doi: 10.1038/s43705-022-00104-9 (PMC9723771; doi:10.1038/s43705-022-00104-9)
Supplement: Supplementary file 6 — Table S6 [file 43705_2022_104_MOESM6_ESM.docx]

The supplementary file S6 can be found on https://github.com/laseab/CC_WR.
